# Current management of cervical cancer in Poland—Analysis of the questionnaire trial for the years 2002-2014 in relation to ASCO 2016 recommendations

**DOI:** 10.1371/journal.pone.0209901

**Published:** 2019-01-31

**Authors:** Tomasz Basta, Paweł Knapp, Paweł Blecharz, Lubomir Bodnar, Iwona Gawron, Dorota Babczyk, Magdalena Piróg, Tomasz Kluz, Anna Markowska, Anna Horbaczewska, Robert Jach

**Affiliations:** 1 Department of Gynecology and Obstetrics, Jagiellonian University Medical College, Krakow, Poland; 2 Department of Gynecology and Gynecological Oncology, Faculty of Medicine, Medical University in Bialystok, Bialystok, Poland; 3 Gynecologic Oncology Department, Centre of Oncology, Maria Sklodowska-Curie Memorial Institute, Krakow Branch, Krakow, Poland; 4 Department of Clinical Oncology, Military Institute of Medicine in Warsaw, Warsaw, Poland; 5 Department of Obstetrics and Gynecology, Fryderyk Chopin University Hospital No 1, Faculty of Medicine, Rzeszow University, Rzeszow, Poland, Poland; 6 Department of Perinatology and Gynecology, Poznan University of Medical Sciences, Poznan, Poland; Rudjer Boskovic Institute, CROATIA

## Abstract

**Objectives:**

To assess the survival of patients with cervical cancer (CC). Since the recommendations concerning cervical cancer management adopted by Polish medical societies do not differ significantly from the ESGO or non-European guidelines, and the fact that evaluation of the system for CC treatment in Poland, as well as the mortality rate of Polish women with CC, which is 70% higher than the average for European Union (EU) countries, justifies the hypothesis that treatment of CC in Poland deviates from the Polish and international recommendations. This article puts forward the current management of cervical cancer in Poland and discusses it in the context of ASCO guidelines.

**Material and methods:**

A survey retrospective multicenter analysis of the medical records of 1247 patients with cervical cancer who underwent treatment for disease and who had completed at least two years of follow-up.

**Results:**

Although concurrent radiotherapy and chemotherapy is a standard treatment of FIGO IB to IVA cervical cancer patients in enhanced- and maximum-resources settings, in our analysis, we found that the percentage of women subjected to chemotherapy was lower than in countries where total survival rates were lower.

**Conclusion:**

Within the IA to II A cervical cancer patients studied group, the methods of treatment remained in line with ASCO guidelines for countries with the highest standard of care.

Although concurrent radiotherapy and chemotherapy is a standard treatment of FIGO IB to IVA cervical cancer patients in enhanced- and maximum-resources settings, in our analysis, we found that the percentage of women subjected to chemotherapy was lower than in countries where total survival rates were lower.

Our findings, together with the inconsistencies within the cervical cancer screening program, may be one of the explanations of poorer survival rate of women with cervical cancer in Poland.

## Introduction

Cervical cancer (CC) is the fourth most common cancer type among women. In 2012, the worldwide number of women with CC was around 528 000, of whom 266 000 died. In 85% of cases, CC is diagnosed in developing countries where it is a leading cause of death among women suffering from cancer [1]. A significant difference in incidence and mortality due to CC is observed between low- and middle-income countries and high-income countries [2, 3, 4].

According to World Bank data, Poland is one of the high-income countries [5]. However, results of CC treatment are unsatisfactory. The GLOBOCAN 2012 project places Poland among countries with the highest rate of incidence and mortality due to CC, with the standardized incidence rate (SIR) at 12.2/100 000 and the standardized death rate (SDR) at 5.4/100,000 (one of the highest among the surveyed countries). Both SIR and CDR are relatively high compared to other European countries [6]. According to the World Health Organization (WHO), therapeutic options in cervical cancer patients should be selected in line with international, national or institutional guidelines, based on the combination of evidence, availability of trained professionals, equipment facilities and infrastructure [7].

In order to provide evidence-based and resource-stratified global recommendations on the management and palliative care of women with invasive CC, the American Society of Clinical Oncology (ASCO) convened a multidisciplinary multinational panel of experts who produced recommendations reflecting settings diversity. The aim was to develop guidelines that would provide the best medical care to patients with CC and, at the same time, could be adapted to four levels of financial resources [8].

Until the 1990s, the standard treatment of CC at the stage of IIB-IVA according to the International Federation of Gynecology and Obstetrics (FIGO), or in earlier stages with histological factors of unfavorable prognosis, was the use of radiotherapy alone. A rapid increase in the incidence of combination therapy (radio- and chemotherapy) has been since the mid 1990s [9]. Extensive multicenter randomized clinical trials (RCTs) [10,11,12] demonstrated extended survival in patients with advanced CC following radiotherapy with concurrent chemotherapy based on cisplatin compared to radiotherapy alone. On the other hand, recommended management of less advanced stages of CC (FIGO I-IIA), including surgery with or without adjuvant therapy, has not undergone any major changes in the last few decades. The recommendations adopted by Polish medical societies [13, 14] do not differ significantly from the FIGO [15] or non-European guidelines [16,17]. Evaluation of the system for CC treatment in Poland [18], as well as the mortality rate of Polish women with CC, which is 70% higher than the average for European Union (EU) countries [19], justifies the hypothesis that treatment of CC in Poland deviates from the Polish and international recommendations. This article puts forward the current management of cervical cancer in Poland and discusses it in the context of ASCO guidelines.

## Material and methods

The retrospective survey study was conducted in the years 2015–2016. The inclusion criteria were women treated due to CC, who had at least two years of post treatment follow-up data. Eligibility of cases excluded factors such as surgical approach, surgery type or primary treatment. Questionnaires were sent to 24 centers of gynecological oncology providing comprehensive treatment of CC. Completed questionnaires were sent back from 16 centers (Szpital Uniwersytecki w Krakowie, Centrum Onkologii im M. Skłodowskiej-Curie, Świętokrzyskie Centrum Onkologii, Wojewódzki Szpital Zespolony w Kielcach, Szpital Specjalistyczny w Brzozowie, Uniwersytecki Szpital Kliniczny w Białymstoku, Białostocki Ośrodek Onkologiczny, Wielkopolskie Centrum Onkologii, Szpital Kliniczny UM w Poznaniu, Szpital Kliniczny w Lublinie, Gdyńskie Centrum Onkologii, DolnoślĄskie Centrum Onkologii, Mazowiecki Szpital Bródnowski, Wojskowy Instytut Medyczny, Szpital Specjalistyczny im. M. Kopernika w Łodzi, Centrum Onkologii w Gliwicach). Of these, 4 centers entered data from 200 patients, 10 centers—25–40 patients, and 2 centers—from 25 patients. The data were collected from 1371 women treated due to CC between 2002 and 2014. The database was designed to capture such data as: clinical staging, primary treatment, complications, imaging, treatment-free survival to the first recurrence, first relapse treatment, complications and imaging associated with this management, treatment-free survival to the second recurrence, second relapse treatment, complications and imaging associated with this management, treatment-free survival to the third recurrence, third relapse treatment, complications and imaging associated with this management, designed to track the treatment of individuals with CC.All data were reviewed by the main and senior author. Any discrepancies in data were corrected through communication between the study monitor and the individual center. Due to the fact that some essential data were not available, 124 patients were excluded from the database. Finally, data from 1247 patients were analyzed. All authors declare no conflict of interest and no financial support. This work was supported by the National Center of Science (NCN grant no. N407 152740).

### Statistical analysis

Data were expressed as a percentage, mean standard deviation or median (interquartile range) unless otherwise stated. The Kolmogorov-Smirnov test was used to assess conformity with a normal distribution.A 2-sided p-value of less than 0.05 was considered statistically significant. Statistical analyses were performed with Statistica 12 (Statsoft, Tulsa, OK, USA).

## Results

Patient age ranged from 27–78, with a median age of 54. The median follow-up after diagnosis was 61 (60–67) months. Among 1218 women, squamous cell cancer was diagnosed in 1070 (87.9%), adenocarcinoma in 112 (9.2%), adenosquamosum in 4 (0.3%) and other types in 32 (2.6%) women. Grading of cervical cancer, based on histopathological examination, was as follows: G1–14 (7.8%), G2–115 (64.3%), and G3–50 (27.9%). The distribution of clinical stage was described in Table 1.

**Table 1:** Clinical stage of cervical cancer according to the FIGO classification.

FIGO stage	N (1145)	(%)
IA	74	6.5
IB	353	30.8
IIA	123	10.7
IIB	289	25.2
IIIA	19	1.7
IIIB	229	20.0
IVA	20	1.8
IVB	38	3.3

### First-line treatment

In the first-line therapy, a surgery was performed in 540 (43%) women (507 cases of radical hysterectomy, (95%); 15 cases of conization, 3% of cases; 11 cases of cervical conization with subsequent radical hysterectomy, 2%. Brachytherapy (BT) was performed in 889 (71%) women and teleradiotherapy (with or without chemotherapy) in 871 (70%) women included in the study (radiotherapy in 194 women, 16%; radiochemotherapy in 677 women, 54%; Detailed analysis of treatment method combinations is presented in Table 2.

**Table 2:** Combinations of treatment methods in the first-line therapy of cervical cancer.

First-line treatment	N (%)	Additional brachytherapy N (% after first-line treatment)	Additional radiotherapy N (% after first-line treatment)	Additional chemotherapy N (% after first-line treatment)
Cc	16 (1.2%)	1 (6.3%)	2 (12.5%)	3 (18.8%)
Cc+RH	10 (0.8%)	1 (10%)	-	-
RH	507 (40.7%)	347 (64.3%)	240 (47.3%)	188 (37.1%)
BT	540 (43.3%)	-	518 (95.9%)	463 (85.7%)
RT	111 (8.9%)	-	-	43 (38.7%)
CT	30 (2.4%)	-	-	-
No treatment	33 (7.7%)	-	-	-

Abbreviations: RH—radical hysterectomy; Cc—cervical conization; BT—brachytherapy; RT—radiotherapy; CT—chemotherapy; RCT—radiochemotherapy;

Chemotherapy was applied in 727 (58.3%) cases. The most commonly used drug was cisplatin alone (50%), less frequently, cisplatin in combination with 5-fluorouracil (21%) and carboplatin with paclitaxel (16%) (Table 3).

**Table 3:** Chemotherapy used in first-line treatment of cervical cancer.

Chemotherapy	N (191)	%
cisplatin	98	51.3
cisplatin and 5-fluorouracil	42	22.0
paclitaxel and carboplatin	31	16.2
cispltin and topotecan	5	2.6
cisplatin and paclitaxel	2	1.0
cisplatin and carboplatin	2	1.0
cisplatin and gemcitabine	1	0.5
topotecan and indicacine	1	0.5
combinations of schemes	9	4.7

Progression after primary treatment was found in 9.3% of cases. Local recurrence and distal metastases occurred in patients treated with brachytherapy in combination with radiotherapy and brachytherapy in combination with radiochemotherapy, and concerned stages FIGO IB and IIB. The type of cervical cancer recurrence was dependent on the first-line treatment. (Table 4)

**Table 4:** Type of cervical cancer recurrence depending on the first-line treatment.

Treatment method	Local recurrence N (42)	Distant metastases N (86)
No treatment	1	2
RH	1	0
RH + BT	3	1
RH + RT	2	0
RH+ RCT	0	2
RH + BT+ RT	2	2
RH + BT+ CT	4	6
RH + BT + RCT	4	6
Cc + BT + RCT	1	0
Cc + RCT	1	0
BT + CT	0	1
BT + RT	1	6
BT	1	0
BT + RCT	17	55
RCT	3	5
RT	1	0

Abbreviations: RH—radical hysterectomy; Cc—cervical conization; BT—brachytherapy; RT—radiotherapy; CT—chemotherapy; RCT—radiochemotherapy;

Distal metastases were more frequent (67.2%) than local recurrence (31%) (Table 5).

**Table 5:** Number of cases of cervical cancer recurrence after first-line treatment in relation to the FIGO stage.

FIGO stage	Local recurrence	Distant metastases
IA	2	0
IB	8	11
IIA	4	15
IIB	15	30
IIIA	2	3
IIIB	7	17
IVA	0	1
IVB	0	3

The highest percentage of local recurrences and distant metastases was found in the group treated with brachytherapy (14.7%) in combination with radiochemotherapy (47.4%) (Table 6), as well as FIGO IIB (12.9%) with distant metastases (25.9%) (Table 7).

**Table 6:** Cervical cancer recurrence treatment (Second-line therapy).

Recurrence treatment	N (116)	%
No further treatment	25	21.5
RH	2	1.7
RH + CT	2	1.7
RH + RCT	1	0.9
Cc + CT	1	0.9
Cc + BT + RCT	1	0.9
BT	3	2.6
BT + RCT	1	0.9
RT	15	12.8
CT	55	47.4
RCT	9	7.8
Cc	1	0.9

Abbreviations: RH—radical hysterectomy; Cc—cervical conization; BT—brachytherapy; RT—radiotherapy; CT—chemotherapy; RCT—radiochemotherapy;

**Table 7:** Chemotherapy used in recurrence treatment.

Treatment	N (76)	(%)
paclitaxel and carboplatin	21	27.6
cisplatin and 5-fluorouracil	17	22.4
cisplatin and topotecan	10	13.2
cisplatin	8	10.5
cisplatin and paclitaxel	6	7.9
paclitaxel	1	1.3
paclitaxel and topotecan	1	1.3
topotecan	1	1.3
ifosfamide	1	1.3
combination of schemes	10	13.2

### Second-line treatment

The first recurrence after primary treatment was most often treated with chemotherapy, i.e. in 55 women (47%). The mean time from the end of primary treatment to the start of recurrence treatment in women included in the study was 62 weeks (95% CI: 50–74 weeks; 15.5 months, 95% CI: 12.5–18.5 months). The most common second-line management types were chemotherapy (47.41%), no treatment (21.6%) and radiotherapy (12.9%) (Table 8).

**Table 8:** Combinations of second-line treatment.

Second-line treatment	N	(%)
No treatment	25	21.5
RH	2	1.7
RH+CT	2	1.7
RH+RCT	1	0.9
Cc+CT	1	0.9
Cc + BT + RCT	1	0.9
BT	3	2.6
BT + RCT	1	0.9
RT	15	12.9
CT	55	47.4
RCT	9	7.8
Cc	1	0.9

Abbreviations: RH—radical hysterectomy; Cc—cervical conization; BT—brachytherapy; RT—radiotherapy; CT—chemotherapy; RCT—radiochemotherapy;

The most frequent regimen used in the second-line chemotherapy was paclitaxel with carboplatin (27%), followed by cisplatin and 5-fluorouracil (22.1%), and cisplatin and topotecan (13%) (Table 9).

**Table 9:** Type of chemotherapy in second-line treatment.

Type of chemotherapy	N	(%)
cisplatin	8	10.4
cisplatin + 5-fluorouracyl	17	22.0
cisplatin + paclitaxel	6	7.8
cisplatin + topotecan	10	13
paclitaxel	1	1.3
paclitaxel + carboplatin	21	27.2
paclitaxel + topotecan	1	1.3
topotecan	1	1.3
ifosfamid	1	1.3
combination	10	13

## Discussion

In the years 2005–2010, the survival rate in CC patients in Poland, according to the National Health Fund (Narodowy Fundusz Zdrowia, NFZ) was 55%. However, according to the National Cancer Registry (Krajowy Rejestr Nowotworów, KRN), in the years 2003–2005, it was 54%, and according to Eurocare, in the years 1999–2007, it was 53%. The values of comparable indicators in Poland were about 10% lower than average levels in Europe (RSC, according to Eurocare) [20]. According to NFZ data, there were significant differences in survival rates between voivodeships, reaching 20 percentage points (Podlaskie Voivodeship 67%, Lodzkie Voivodeship 45%). There were also discrepancies in these indicators depending on the data source (NFZ data vs. KRN data), which cannot be explained by other data methodology [21]. Treatment of CC at an early stage involves surgery and radiotherapy. Surgical methods are generally reserved for stage IA, IB1, and in some cases, IIA1.

Chemoradiotherapy is a method of choice in the treatment of stages IB2 to IVA. It can also be used in patients who are not eligible for surgery [22, 23]. In guidelines of leading societies (with the exception of the European Society for Medical Oncology- ESMO), radical hysterectomy with pelvic lymphadenectomy, and para-aortic lymph node sampling in selected cases, are recommended in women with an early-stage or locally advanced disease. According to ESMO, radical hysterectomy is not recommended for the early stage of the disease. Microinvasive cervical cancer (stage IA1) without lymphovascular space invasion (LVSI) can be managed with conization or simple trachelectomy in order to preserve fertility, and simple hysterectomy can be offered if the patient does not wish to preserve fertility. In stage IA1 with LVSI, surgical assessment of pelvic lymph nodes should be discussed with the patient, including the sentinel lymph node. This approach provides a much less mutilating procedure for the patient [24, 25, 26].In our study, we found that in the first-line therapy, surgery was performed in 540 (43%) patients (507 cases of radical hysterectomy, 95%; 15 cases of conization, 3% of cases; and 11 cases of cervical conization with subsequent radical hysterectomy, 2%. Taking into account that the stage IA to IIA group accounted for 48% of patients, this method of treatment remained in line with ASCO guidelines for countries with the highest standard of care. For women with locally advanced CC, the role of surgery has been debated for many years, and little benefit from such management has been found for most women in stages IB2 to III [27]. Concurrent radiotherapy and chemotherapy is a standard treatment in FIGO IB to IVA in enhanced- and maximum-resources settings [28, 29, 30, 31, 32, 33, 34].

In our analysis, we found that brachytherapy was performed in 71% and teleradiotherapy (with or without chemotherapy) in 70% women included in the study (radiotherapy in 16%; radiochemotherapy in 54%). Chemotherapy was applied in 58% cases. The most commonly used drug was cisplatin (50% of cases), less frequently, cisplatin in combination with 5-fluorouracil (21%) and carboplatin with paclitaxel (16%). In our study population, the percentage of women subjected to chemotherapy was lower than in countries where total survival rates are lower [35]. Perhaps, this observation resulted from the fact that attempts were made not to delay the use of radiotherapy as a result of administrating chemotherapy. In 16% of women, the chemotherapy scheme consisted of carboplatin and paclitaxel, which proves the management of high-income settings, as shown in the ongoing clinical trial [36]. The purpose of this trial is to investigate the influence of the addition of adjuvant carboplatin and paclitaxel after chemoradiotherapy versus concurrent cisplatin and radiotherapy in FIGO IB to IVA patients. An additional phase III trial of concurrent chemotherapy and pelvic irradiation with or without adjuvant carboplatin and paclitaxel is open for high-risk patients with stage IA2, IB, or IIA cervical carcinoma after radical hysterectomy [37].

### Strengths of the study

This was the first study investigating CC management in the Polish population. The observation time in this study was longer than that reported in other studies [38], nevertheless, the mean follow- up was 5.5 years.

### Limitations of the study

As other population studies based on administrative data [39,40], this study has some limitations related to data availability. Therefore, we were only able to assess tumor-related factors, management and recurrence-free survival. We were not able to consider additional individual factors, in particular, the effects of comorbidities, general health condition and preferences in the treatment. Similarly, we were not able to evaluate other factors related to medical practice, such as local practice of referring patients to an oncology center, potential limitations in healthcare access or patient’s adherence to treatment. Therefore, we have no certainty how each of these uncontrolled factors contributed to the observed outcomes. Tracing the extent to which clinical practice coincides with recommendations is a matter to be considered in future research.

## Conclusions

Within the studied group of IA to II A cervical cancer patients, the methods of treatment remained in line with ASCO guidelines for countries with the highest standard of care. Although concurrent radiotherapy and chemotherapy is a standard treatment of FIGO IB to IVA cervical cancer patients in enhanced- and maximum-resources settings, in our analysis, we found that the percentage of women subjected to chemotherapy was lower than in countries where total survival rates were lower.

Our findings, together with the inconsistencies within the cervical cancer screening program, may be one of the explanations of poorer survival rate of women with cervical cancer in Poland.

## Supporting information

S1 File English version of the questionnaire conducted in the centres of gynecological oncology. (DOCX)

S2 File Polish version of the questionnaire conducted in the centres of gynecological oncology. (PDF)

S3 File Manuscript with track changes. (DOCX)

S4 File Data collected from the centres of gynecological oncology. (DOC)
