# Supplementary material for: Current management of cervical cancer in Poland—Analysis of the questionnaire trial for the years 2002-2014 in relation to ASCO 2016 recommendations
Source: PLoS One. 2019 Jan 31;14(1):e0209901. doi: 10.1371/journal.pone.0209901 (PMC6354992; doi:10.1371/journal.pone.0209901)
Supplement: S1 File — (DOCX) [file pone.0209901.s001.docx]

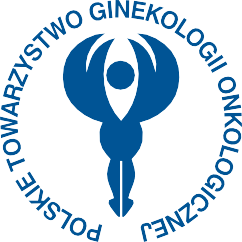
**DAILY DIARY STUDY
OF CERVICAL CANCER**


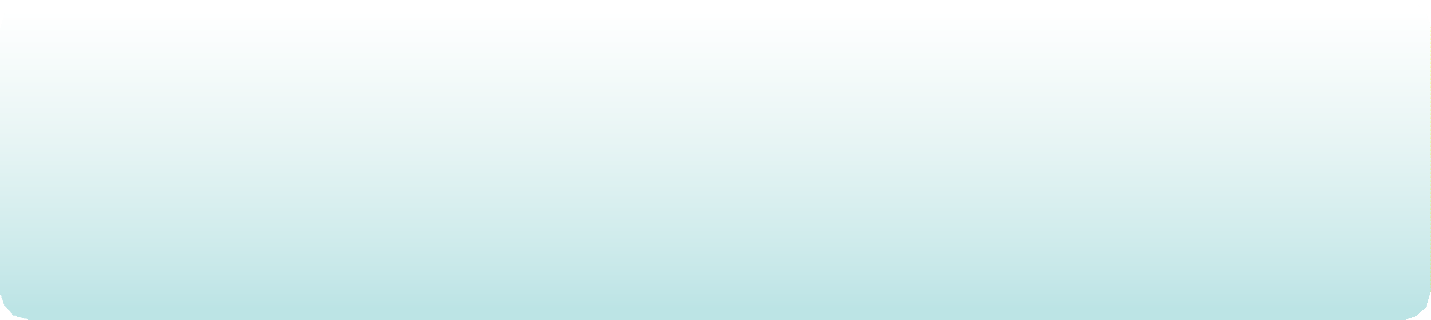

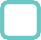


STAGE OF DEVELOPMENT

IA ❑ IB ❑ IIA ❑ IIB ❑ IIIA ❑ IIIB ❑ IVA ❑ IVB ❑

HISTOLOGICAL TYPE OF CANCER: . . . . . . . . . . . . . . . . . . . . . . . . . . . . . . . . . . . . . . . . . . . . . . . . . . . . . . . . . . . . . . . . . . . . .

CONCOMITANT PREGNANCY (during and up to 12 months after the conclusion of treatment) YES ❑ NO ❑

DRG CLASSIFICATION: . . . . . . . . . . . . . . .

**2**


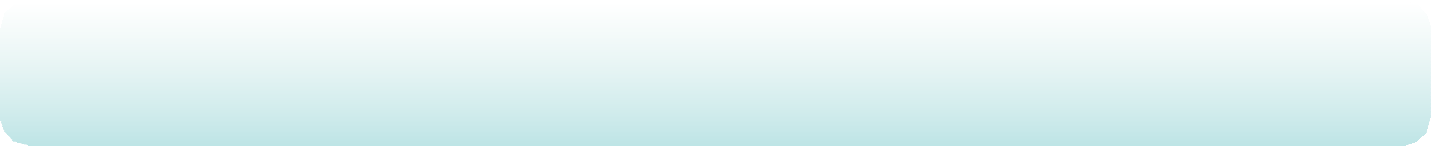

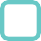


CENTRE AND PATIENT NUMBER

Centre no.

Patient no.

**1**

TYPE OF PRIMARY TREATMENT


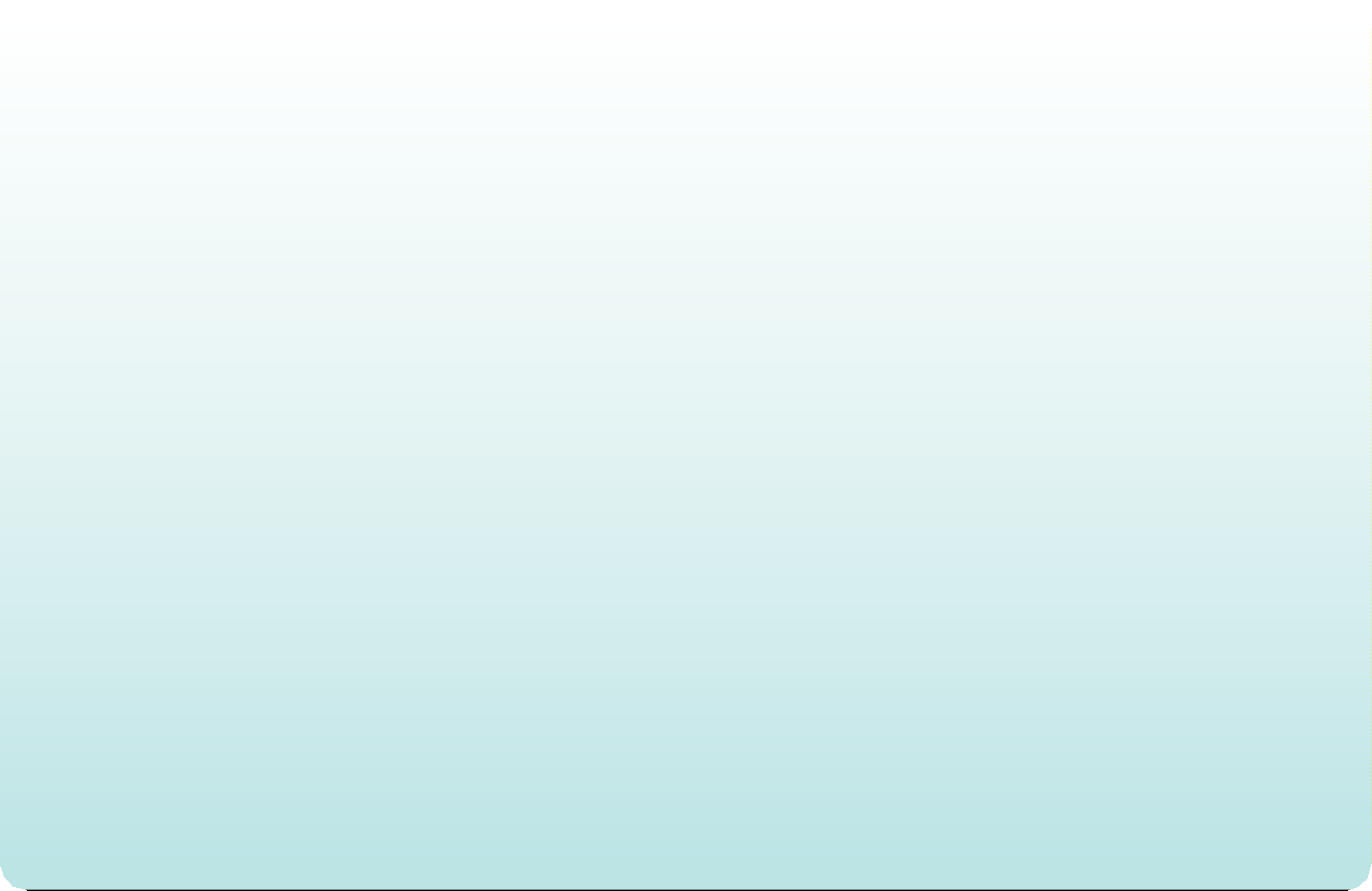

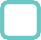


❑ Surgical procedure

LEEP/LLETZ ❑ electroconization ❑ conization ❑ hysterectomy ❑

TIME SPENT IN THE UNIT: days

TIME SPENT IN THE OPERATING THEATRE: min.

❑ Brachytherapy

❑ EBT:

❑ CHT:

combined radiation and chemotherapy ❑ radiation therapy ❑

type of chemotherapy . . . . . . . . . . . . . . . . . . . . . . . . . . . . . . . . . . . . . . . . . . . . . . . . . . . . .

. . . . . . . . . . . . . . . . . . . . . . . . . . . . . . . . . . . . . . . . . . . . . . . . . . . . . . . . . . . . . . . . . . . . . . . .

. . . . . . . . . . . . . . . .

number of cycles: . . . . . . . . . .

reasons for discontinuation of treatment:

remission ❑ adverse reactions ❑ stabilization ❑ progression ❑

Date of beginning of primary treatment: . . . . . . . . . . . . . . . . . . . . .


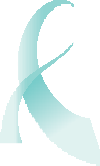

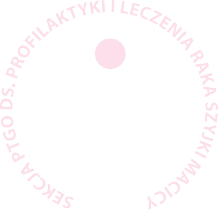


Date of conclusion of primary treatment: . . . . . . . . . . . . . . . . . . . . . **3**

COMPLICATIONS


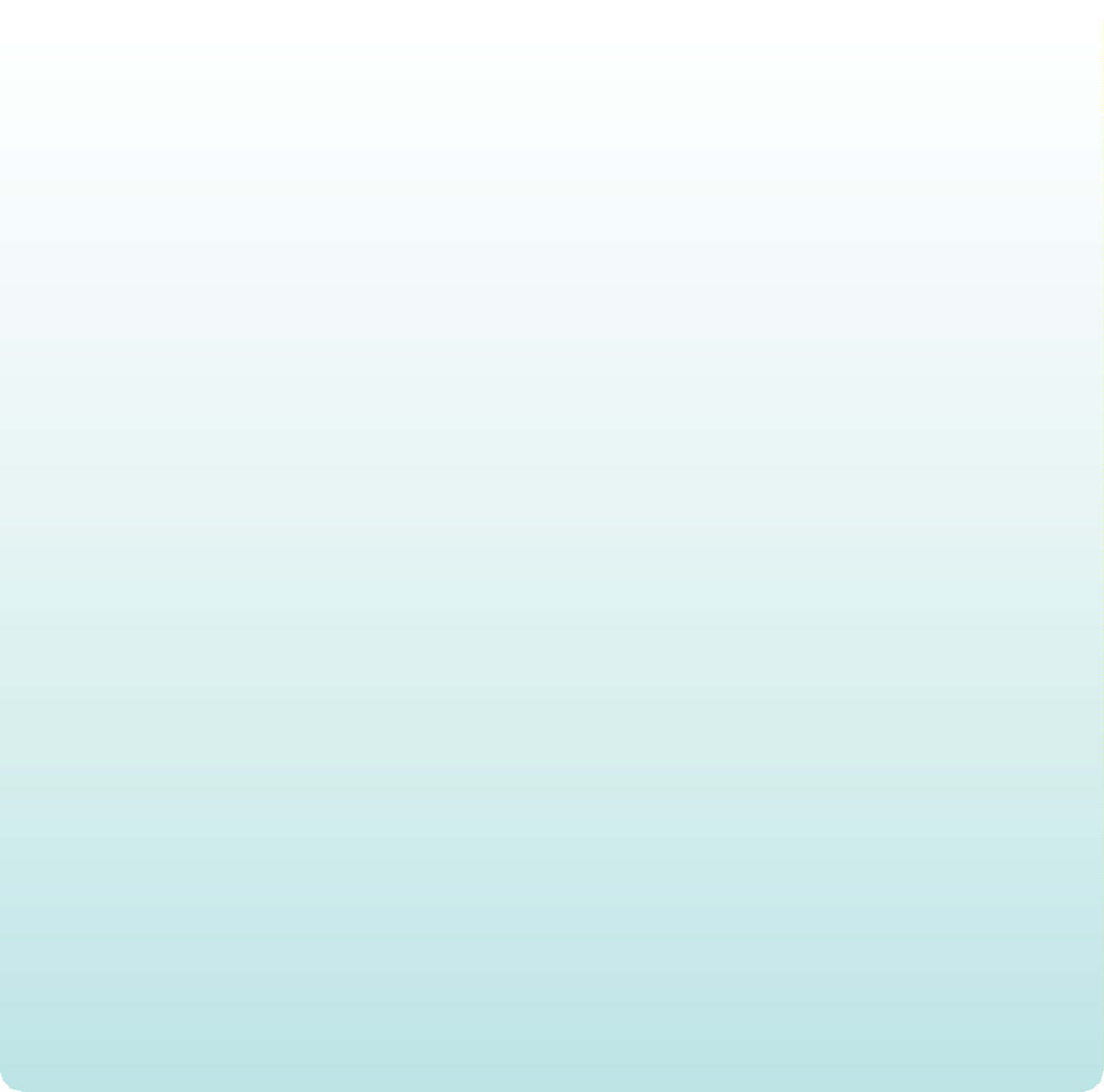

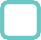


❑ fistula

type of fistula: . . . . . . . . . . . . . . . . . . . . . . . . . . . . . . . . . . . . . . . . . . . . . . . . . . . . . . . . . . . . . .

date of fistula surgery: . . . . . . . . . . . . . . .

❑ haematological complications

treatment applied: . . . . . . . . . . . . . . . . . . . . . . . . . . . . . . . . . . . . . . . . . . . . . . . . . . . . . . . . . .

. . . . . . . . . . . . . . . . . . . . . . . . . . . . . . . . . . . . . . . . . . . . . . . . . . . . . . . . . . . . . . . . . . . . . . . . .

❑ nephrological complications

treatment applied: . . . . . . . . . . . . . . . . . . . . . . . . . . . . . . . . . . . . . . . . . . . . . . . . . . . . . . . . . .

. . . . . . . . . . . . . . . . . . . . . . . . . . . . . . . . . . . . . . . . . . . . . . . . . . . . . . . . . . . . . . . . . . . . . . . . .

❑ hepatic complications

treatment applied: . . . . . . . . . . . . . . . . . . . . . . . . . . . . . . . . . . . . . . . . . . . . . . . . . . . . . . . . . .

. . . . . . . . . . . . . . . . . . . . . . . . . . . . . . . . . . . . . . . . . . . . . . . . . . . . . . . . . . . . . . . . . . . . . . . . .

❑ cardiovascular complications

treatment applied: . . . . . . . . . . . . . . . . . . . . . . . . . . . . . . . . . . . . . . . . . . . . . . . . . . . . . . . . . .

. . . . . . . . . . . . . . . . . . . . . . . . . . . . . . . . . . . . . . . . . . . . . . . . . . . . . . . . . . . . . . . . . . . . . . . . .

❑ neurological complications

treatment applied: . . . . . . . . . . . . . . . . . . . . . . . . . . . . . . . . . . . . . . . . . . . . . . . . . . . . . . . . . .

. . . . . . . . . . . . . . . . . . . . . . . . . . . . . . . . . . . . . . . . . . . . . . . . . . . . . . . . . . . . . . . . . . . . . . . . .

❑ gastrointestinal complications

treatment applied: . . . . . . . . . . . . . . . . . . . . . . . . . . . . . . . . . . . . . . . . . . . . . . . . . . . . . . . . . .

. . . . . . . . . . . . . . . . . . . . . . . . . . . . . . . . . . . . . . . . . . . . . . . . . . . . . . . . . . . . . . . . . . . . . . . . .

❑ lymphedema

treatment applied: . . . . . . . . . . . . . . . . . . . . . . . . . . . . . . . . . . . . . . . . . . . . . . . . . . . . . . . . . .

. . . . . . . . . . . . . . . . . . . . . . . . . . . . . . . . . . . . . . . . . . . . . . . . . . . . . . . . . . . . . . . . . . . . . . . . .

**4**


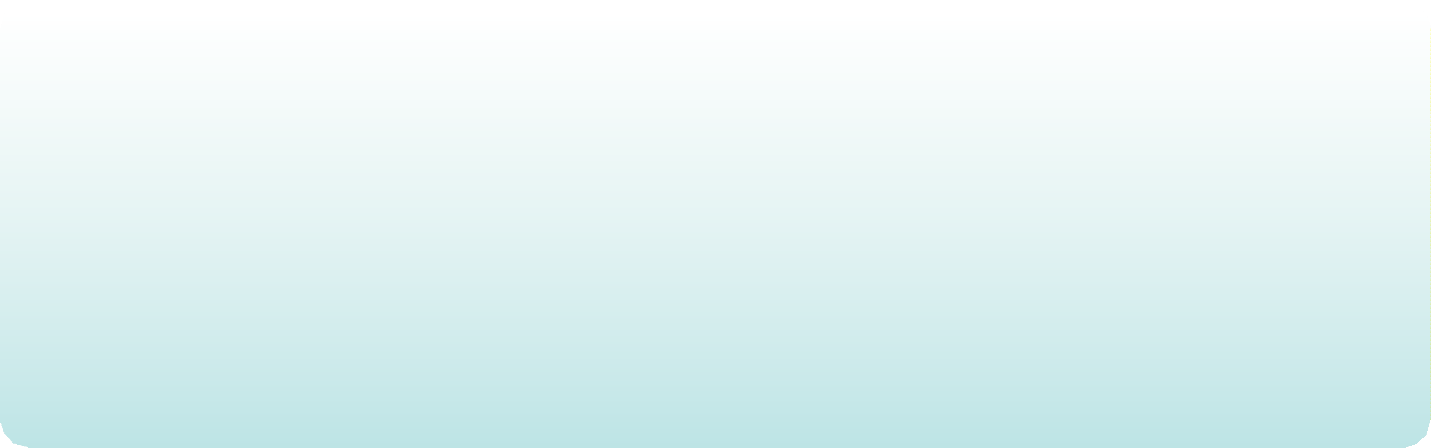

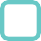


IMAGING TESTS DONE DURING DIAGNOSIS AND PRIMARY TREATMENT

❑ CT

❑ Ultrasonography

❑ MRI

❑ Scintigraphy

❑ PET

❑ Colposcopy

number: . . . . . . . .

number: . . . . . . . .

number: . . . . . . . .

number: . . . . . . . .

number: . . . . . . . .

number: . . . . . . . .

**5**


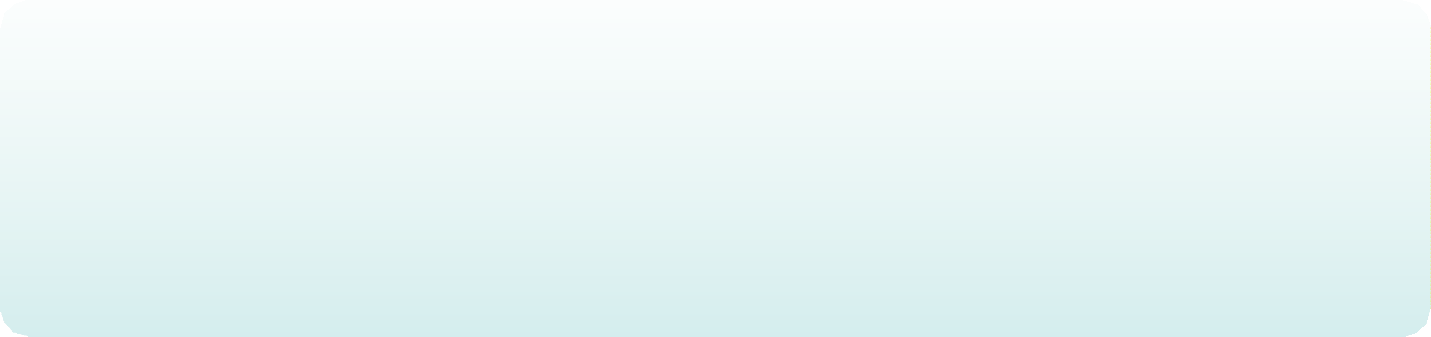

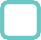


TREATMENT FREE SURVIVAL (INTERVAL BETWEEN THE CONCLUSION OF PRIMARY TREATMENT AND THE BEGINNING

OF TREATMENT OF RELAPSE):

. . . . . . . . . . . . . . . . . . . . . . . . . . . . . (weeks)

TYPE OF PROGRESSION:

❑ local relapse

❑ distant metastasis

**6**


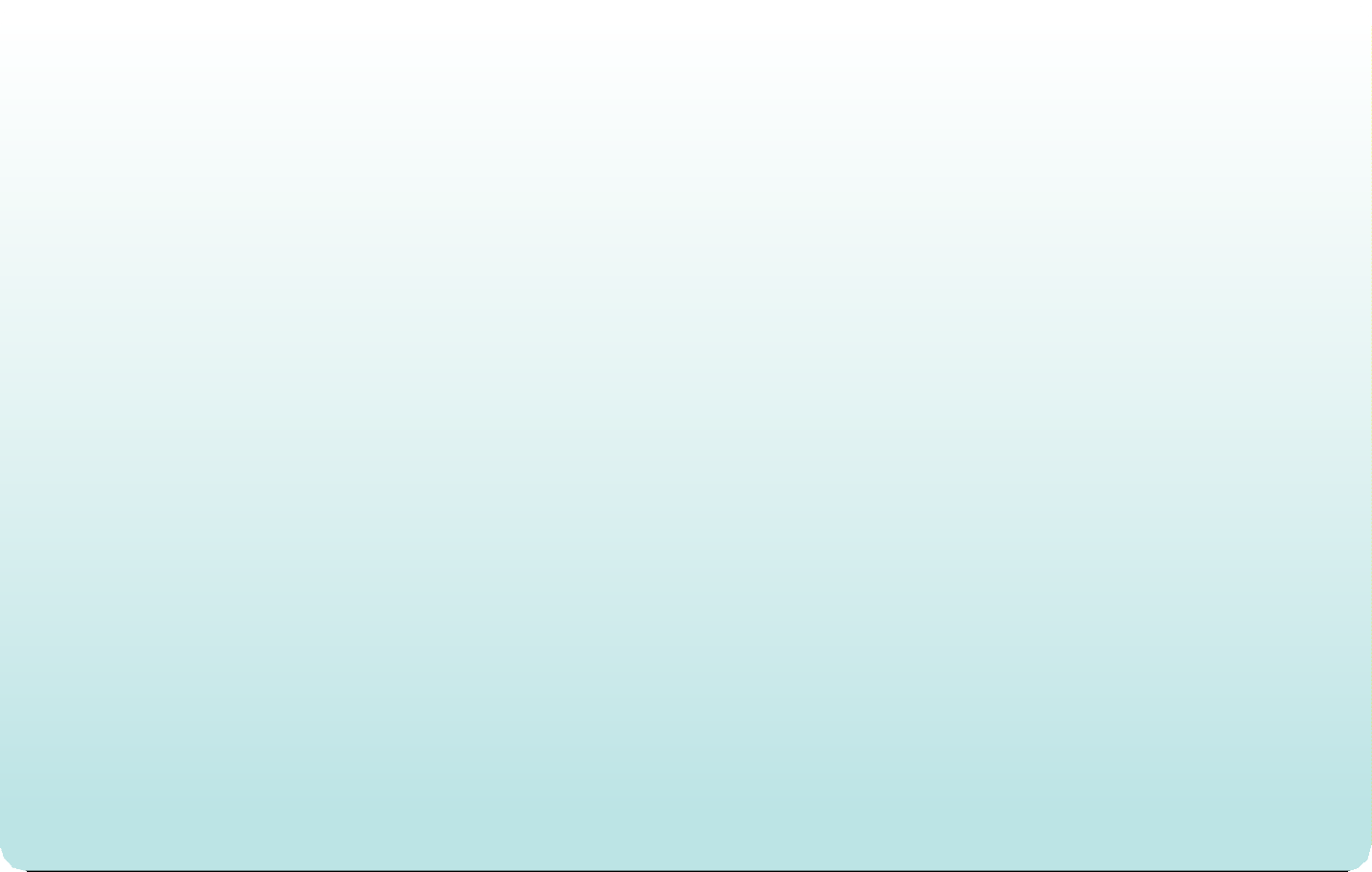

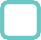


TYPE OF TREATMENT OF 1ST RELAPSE

❑ Surgical procedure

LEEP/LLETZ ❑ electroconization ❑ conization ❑ hysterectomy ❑

TIME SPENT IN THE UNIT: days

TIME SPENT IN THE OPERATING THEATRE: min.

❑ Brachytherapy

❑ EBT:

❑ CHT:

combined radiation and chemotherapy ❑ radiation therapy ❑

type of chemotherapy . . . . . . . . . . . . . . . . . . . . . . . . . . . . . . . . . . . . . . . . . . . . . . . . . . . .

. . . . . . . . . . . . . . . . . . . . . . . . . . . . . . . . . . . . . . . . . . . . . . . . . . . . . . . . . . . . . . . . . .

. . . . . . . . . . . . . . . .

number of cycles: . . . . . . . . . .

reasons for discontinuation of treatment:

remission ❑ adverse reactions ❑ stabilization ❑ progression ❑

Date of beginning of treatment of 1st relapse: . . . . . . . . . . . . . . . . . . . . .


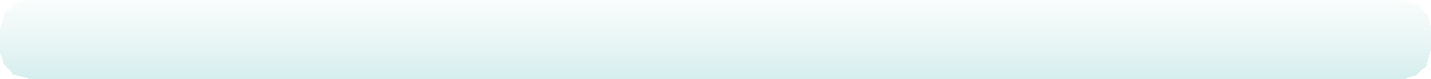


**TREATMENT OF 1ST RELAPSE**

Date of conclusion of treatment of 1st relapse: . . . . . . . . . . . . . . . . . . . . . **7**

COMPLICATIONS


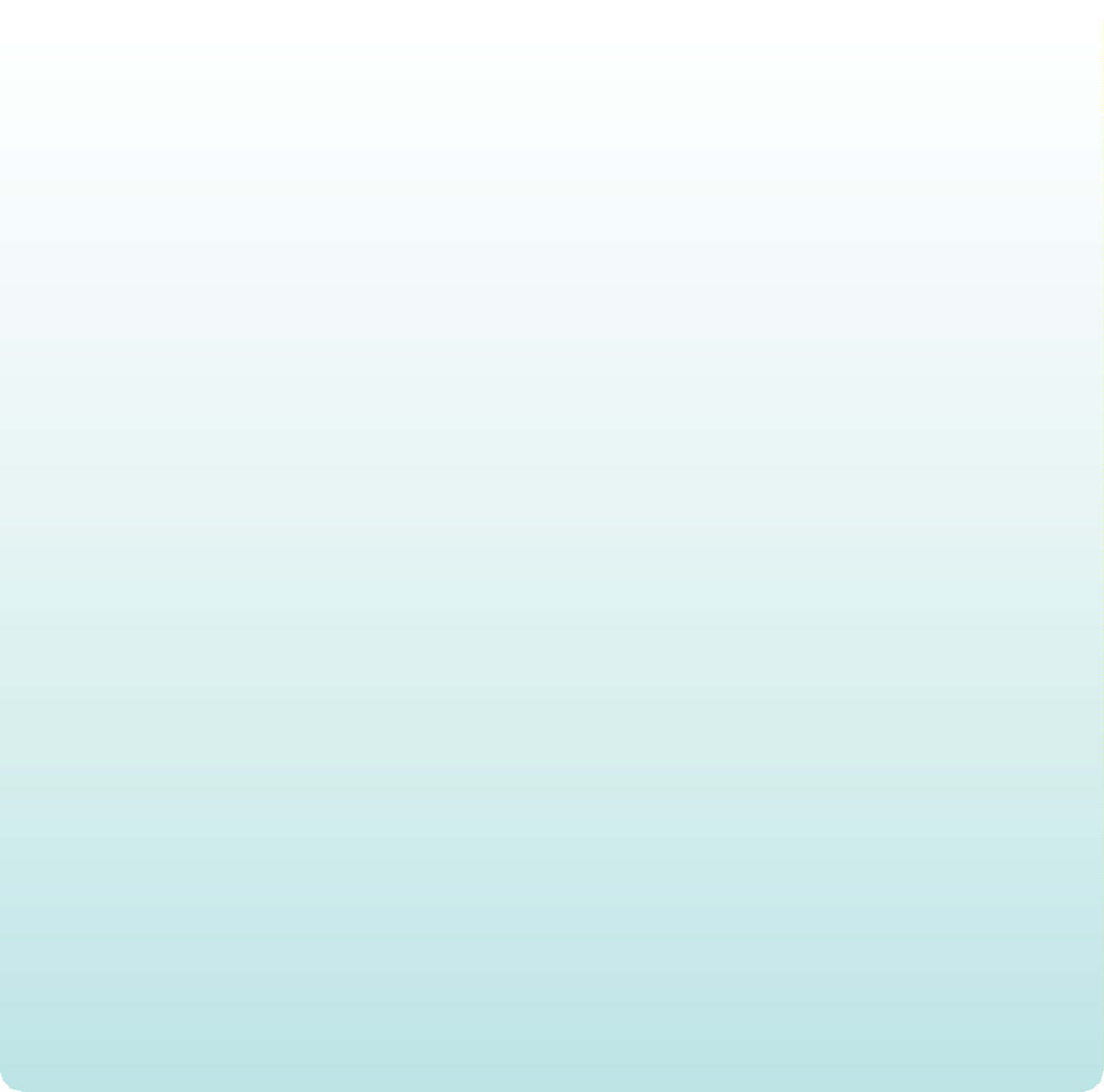

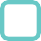


❑ fistula

type of fistula: . . . . . . . . . . . . . . . . . . . . . . . . . . . . . . . . . . . . . . . . . . . . . . . . . . . . . . . . . . . . . .

date of fistula surgery: . . . . . . . . . . . . . . .

❑ haematological complications

treatment applied: . . . . . . . . . . . . . . . . . . . . . . . . . . . . . . . . . . . . . . . . . . . . . . . . . . . . . . . . . .

. . . . . . . . . . . . . . . . . . . . . . . . . . . . . . . . . . . . . . . . . . . . . . . . . . . . . . . . . . . . . . . . . . . . . . . . .

❑ nephrological complications

treatment applied: . . . . . . . . . . . . . . . . . . . . . . . . . . . . . . . . . . . . . . . . . . . . . . . . . . . . . . . . . .

. . . . . . . . . . . . . . . . . . . . . . . . . . . . . . . . . . . . . . . . . . . . . . . . . . . . . . . . . . . . . . . . . . . . . . . . .

❑ hepatic complications

treatment applied: . . . . . . . . . . . . . . . . . . . . . . . . . . . . . . . . . . . . . . . . . . . . . . . . . . . . . . . . . .

. . . . . . . . . . . . . . . . . . . . . . . . . . . . . . . . . . . . . . . . . . . . . . . . . . . . . . . . . . . . . . . . . . . . . . . . .

❑ cardiovascular complications

treatment applied: . . . . . . . . . . . . . . . . . . . . . . . . . . . . . . . . . . . . . . . . . . . . . . . . . . . . . . . . . .

. . . . . . . . . . . . . . . . . . . . . . . . . . . . . . . . . . . . . . . . . . . . . . . . . . . . . . . . . . . . . . . . . . . . . . . . .

❑ neurological complications

treatment applied: . . . . . . . . . . . . . . . . . . . . . . . . . . . . . . . . . . . . . . . . . . . . . . . . . . . . . . . . . .

. . . . . . . . . . . . . . . . . . . . . . . . . . . . . . . . . . . . . . . . . . . . . . . . . . . . . . . . . . . . . . . . . . . . . . . . .

❑ gastrointestinal complications

treatment applied: . . . . . . . . . . . . . . . . . . . . . . . . . . . . . . . . . . . . . . . . . . . . . . . . . . . . . . . . . .

. . . . . . . . . . . . . . . . . . . . . . . . . . . . . . . . . . . . . . . . . . . . . . . . . . . . . . . . . . . . . . . . . . . . . . . . .

❑ lymphedema

treatment applied: . . . . . . . . . . . . . . . . . . . . . . . . . . . . . . . . . . . . . . . . . . . . . . . . . . . . . . . . . .

. . . . . . . . . . . . . . . . . . . . . . . . . . . . . . . . . . . . . . . . . . . . . . . . . . . . . . . . . . . . . . . . . . . . . . . . .

**8**


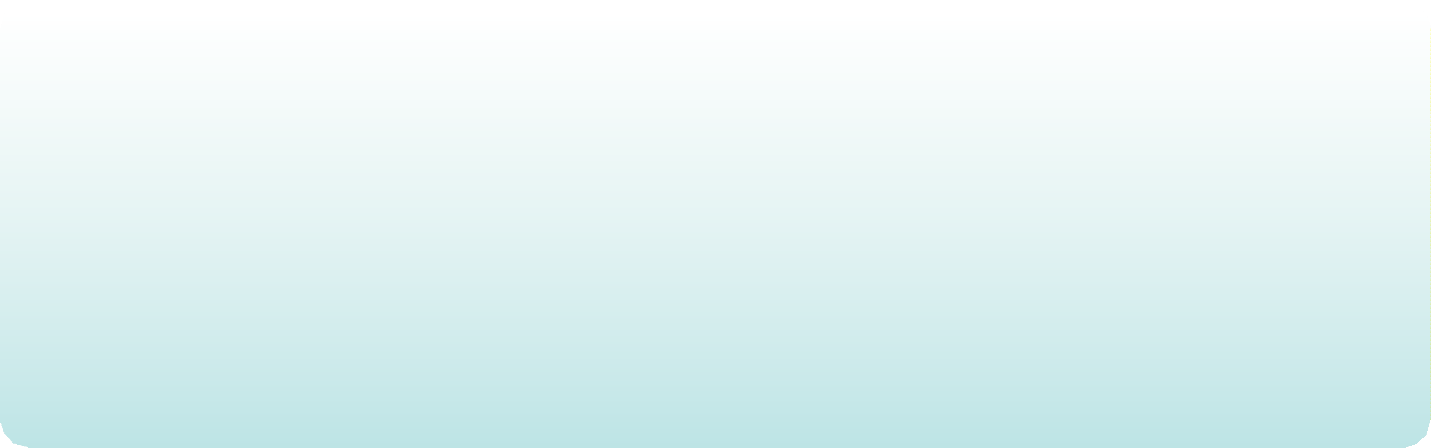

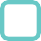


IMAGING TESTS DONE DURING DIAGNOSIS AND TREATMENT OF RELAPSE

❑ CT

❑ Ultrasonography

❑ MRI

❑ Scintigraphy

❑ PET

❑ Colposcopy

number: . . . . . . . .

number: . . . . . . . .

number: . . . . . . . .

number: . . . . . . . .

number: . . . . . . . .

number: . . . . . . . .

**9**


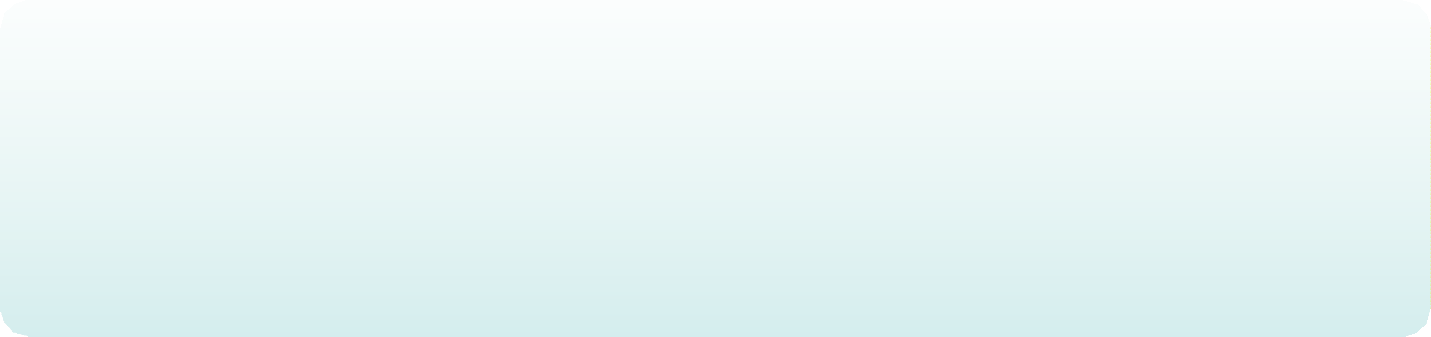

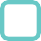


TREATMENT FREE SURVIVAL (INTERVAL BETWEEN THE CONCLUSION OF TREATMENT OF 1ST RELAPSE AND THE BEGINNING OF TREATMENT OF 2ND RELAPSE):

. . . . . . . . . . . . . . . . . . . . . . . . . . . . . (weeks)

TYPE OF PROGRESSION:

❑ local relapse

❑ distant metastasis

**10**


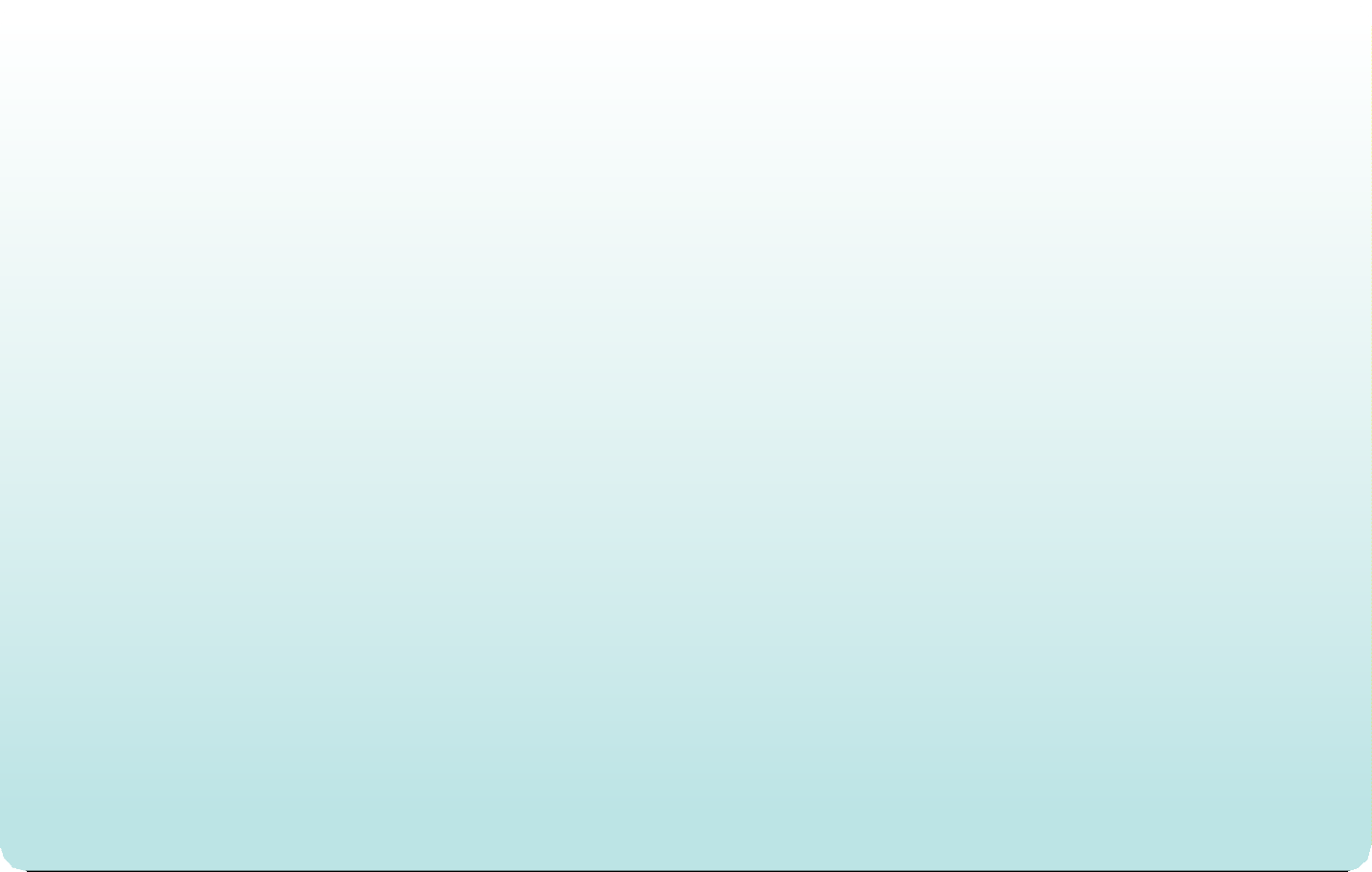

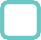


TYPE OF TREATMENT OF 2ND RELAPSE

❑ Surgical procedure

LEEP/LLETZ ❑ electroconization ❑ conization ❑ hysterectomy ❑

TIME SPENT IN THE UNIT: days

TIME SPENT IN THE OPERATING THEATRE: min.

❑ Brachytherapy

❑ EBT:

❑ CHT:

combined radiation and chemotherapy ❑ radiation therapy ❑

type of chemotherapy . . . . . . . . . . . . . . . . . . . . . . . . . . . . . . . . . . . . . . . . . . . . . . . . . . . .

. . . . . . . . . . . . . . . . . . . . . . . . . . . . . . . . . . . . . . . . . . . . . . . . . . . . . . . . . . . . . . . . . .

. . . . . . . . . . . . . . . .

number of cycles: . . . . . . . . . .

reasons for discontinuation of treatment:
remission ❑ adverse reactions ❑ stabilization ❑ progression ❑

Date of beginning of treatment of 2nd relapse: . . . . . . . . . . . . . . . . . . . . .


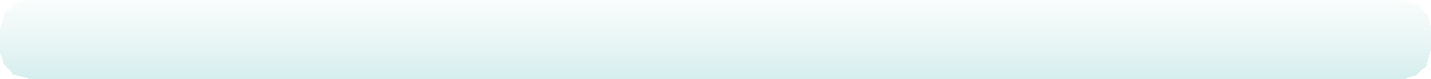


**TREATMENT OF 2ND RELAPSE**

Date of conclusion of treatment of 2nd relapse: . . . . . . . . . . . . . . . . . . . . . **11**

COMPLICATIONS


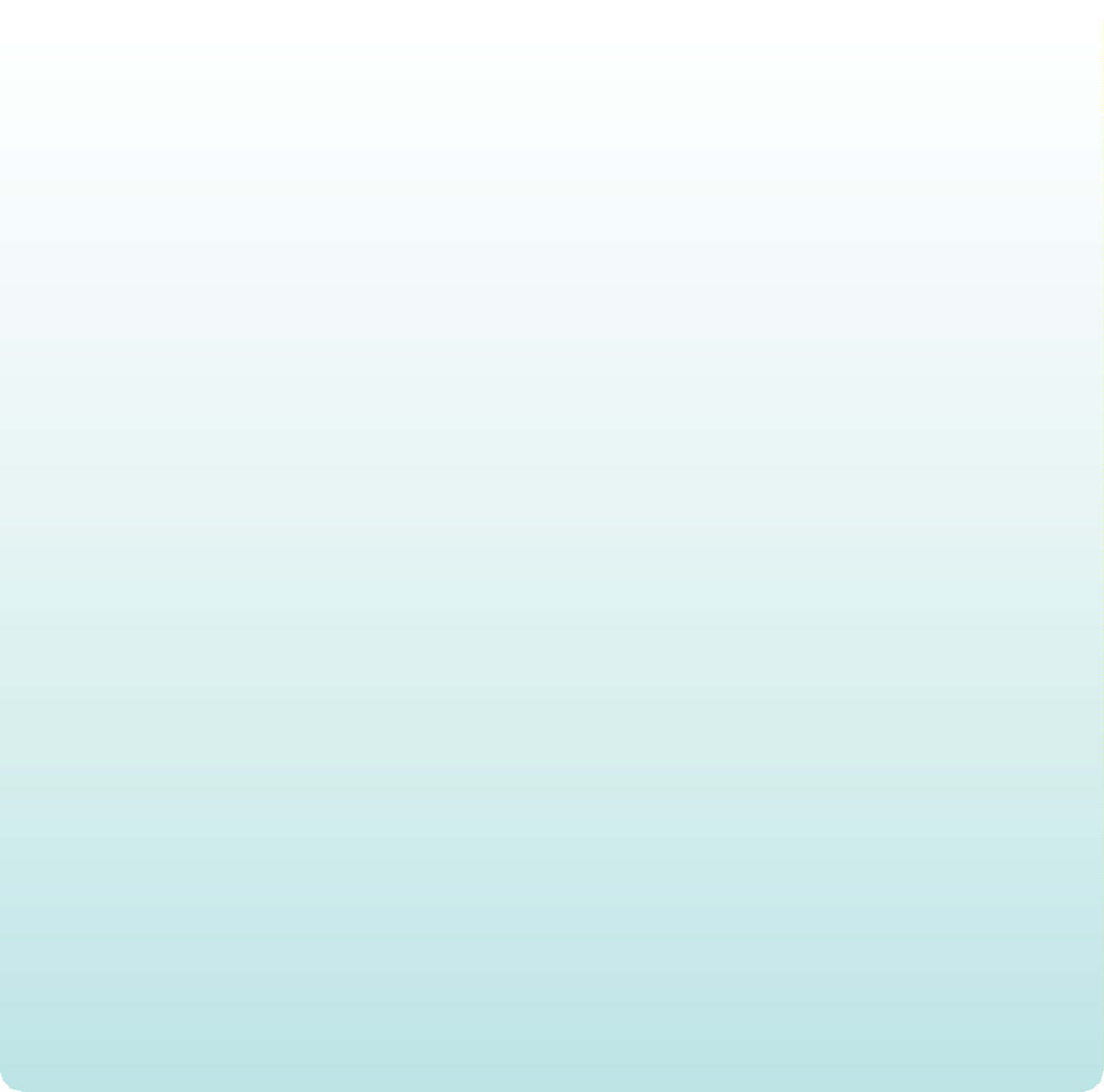

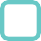


❑ fistula

type of fistula: . . . . . . . . . . . . . . . . . . . . . . . . . . . . . . . . . . . . . . . . . . . . . . . . . . . . . . . . . . . . . .

date of fistula surgery: . . . . . . . . . . . . . . .

❑ haematological complications

treatment applied: . . . . . . . . . . . . . . . . . . . . . . . . . . . . . . . . . . . . . . . . . . . . . . . . . . . . . . . . . .

. . . . . . . . . . . . . . . . . . . . . . . . . . . . . . . . . . . . . . . . . . . . . . . . . . . . . . . . . . . . . . . . . . . . . . . . .

❑ nephrological complications

treatment applied: . . . . . . . . . . . . . . . . . . . . . . . . . . . . . . . . . . . . . . . . . . . . . . . . . . . . . . . . . .

. . . . . . . . . . . . . . . . . . . . . . . . . . . . . . . . . . . . . . . . . . . . . . . . . . . . . . . . . . . . . . . . . . . . . . . . .

❑ hepatic complications

treatment applied: . . . . . . . . . . . . . . . . . . . . . . . . . . . . . . . . . . . . . . . . . . . . . . . . . . . . . . . . . .

. . . . . . . . . . . . . . . . . . . . . . . . . . . . . . . . . . . . . . . . . . . . . . . . . . . . . . . . . . . . . . . . . . . . . . . . .

❑ cardiovascular complications

treatment applied: . . . . . . . . . . . . . . . . . . . . . . . . . . . . . . . . . . . . . . . . . . . . . . . . . . . . . . . . . .

. . . . . . . . . . . . . . . . . . . . . . . . . . . . . . . . . . . . . . . . . . . . . . . . . . . . . . . . . . . . . . . . . . . . . . . . .

❑ neurological complications

treatment applied: . . . . . . . . . . . . . . . . . . . . . . . . . . . . . . . . . . . . . . . . . . . . . . . . . . . . . . . . . .

. . . . . . . . . . . . . . . . . . . . . . . . . . . . . . . . . . . . . . . . . . . . . . . . . . . . . . . . . . . . . . . . . . . . . . . . .

❑ gastrointestinal complications

treatment applied: . . . . . . . . . . . . . . . . . . . . . . . . . . . . . . . . . . . . . . . . . . . . . . . . . . . . . . . . . .

. . . . . . . . . . . . . . . . . . . . . . . . . . . . . . . . . . . . . . . . . . . . . . . . . . . . . . . . . . . . . . . . . . . . . . . . .

❑ lymphedema

treatment applied: . . . . . . . . . . . . . . . . . . . . . . . . . . . . . . . . . . . . . . . . . . . . . . . . . . . . . . . . . .

. . . . . . . . . . . . . . . . . . . . . . . . . . . . . . . . . . . . . . . . . . . . . . . . . . . . . . . . . . . . . . . . . . . . . . . . .

**12**


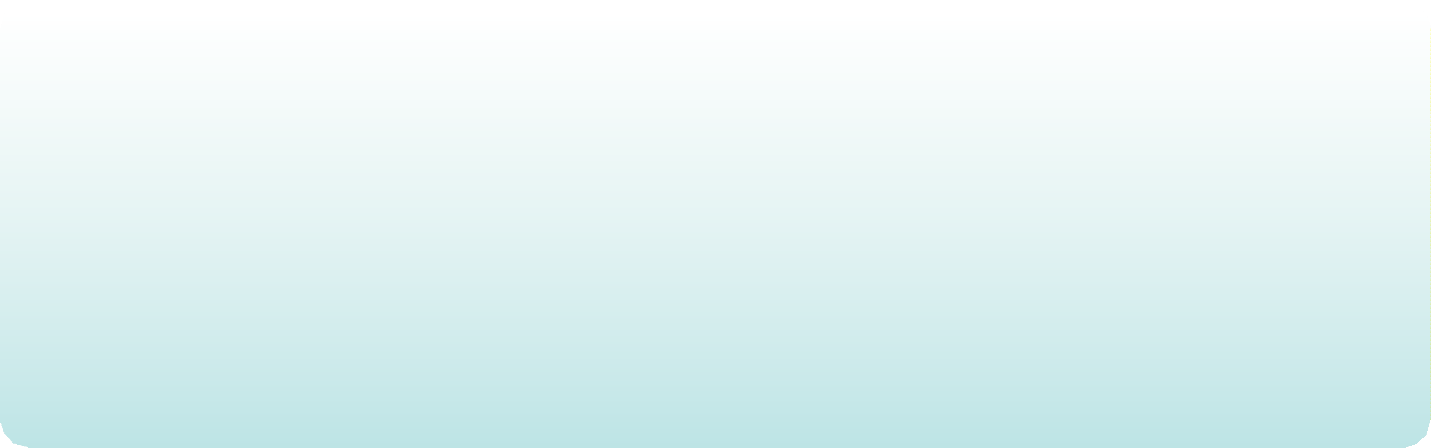

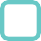


IMAGING TESTS DONE DURING DIAGNOSIS AND TREATMENT OF 2ND RELAPSE

❑ CT

❑ Ultrasonography

❑ MRI

❑ Scintigraphy

❑ PET

❑ Colposcopy

number: . . . . . . . .

number: . . . . . . . .

number: . . . . . . . .

number: . . . . . . . .

number: . . . . . . . .

number: . . . . . . . .

**13**


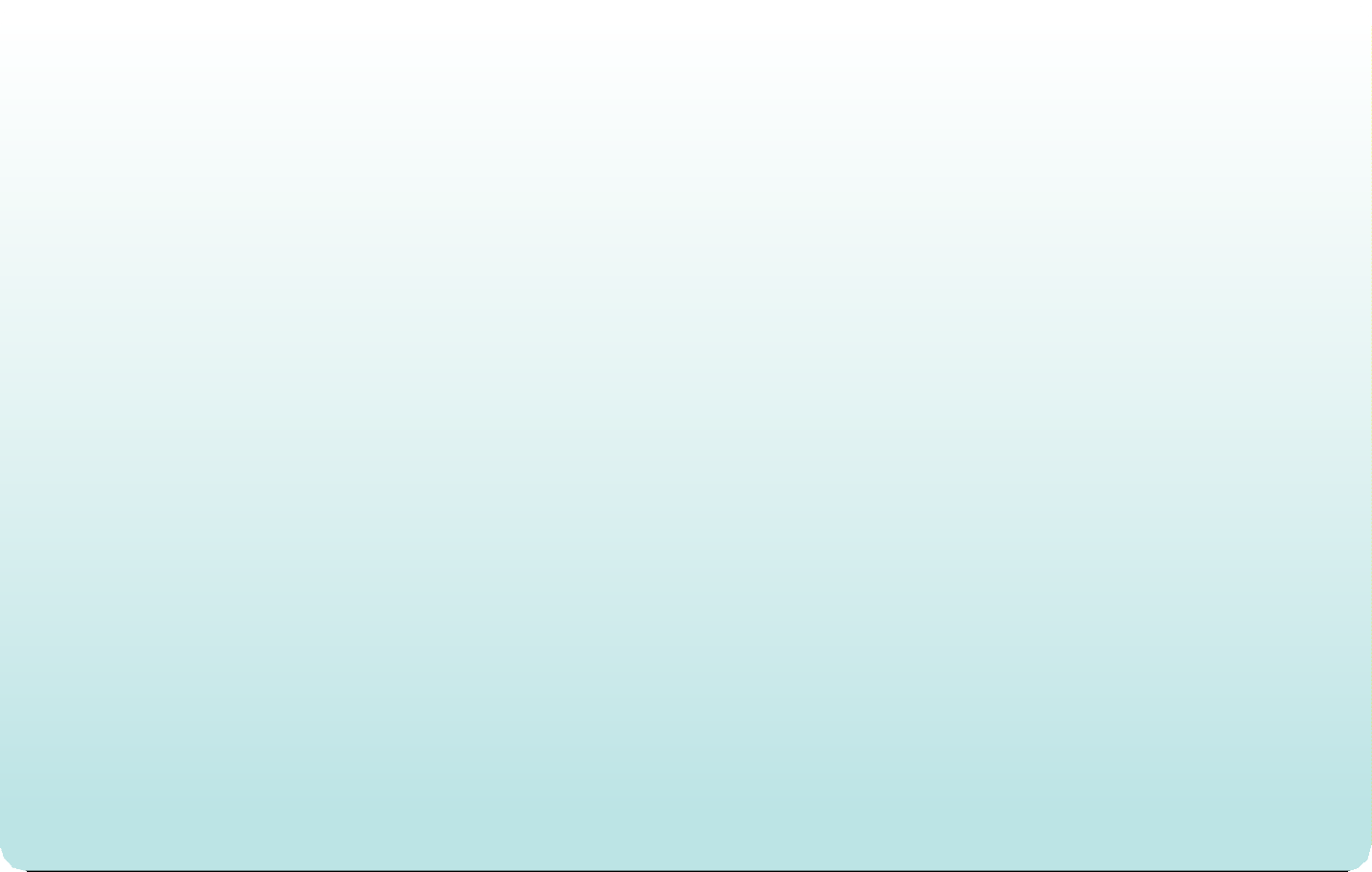

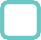


TYPE OF TREATMENT OF 3RD RELAPSE


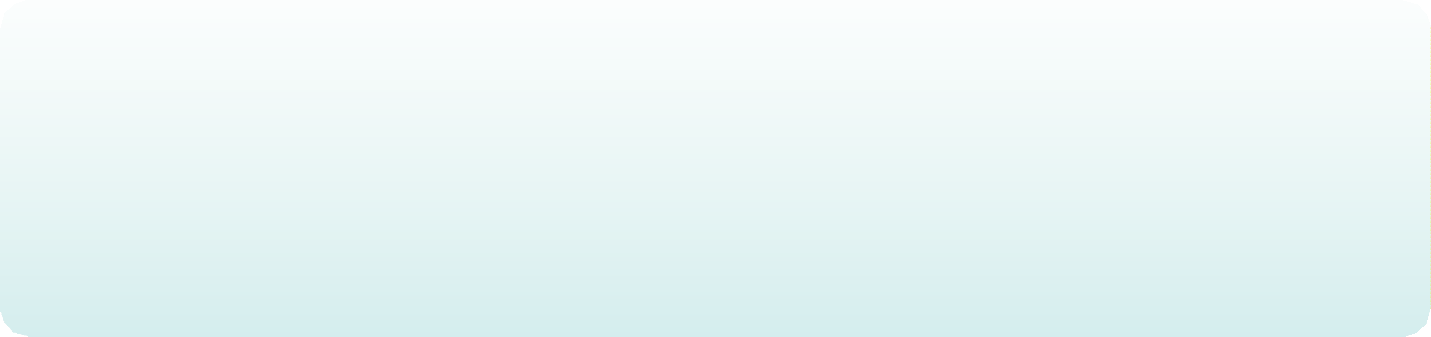

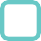


TREATMENT FREE SURVIVAL (INTERVAL BETWEEN THE CONCLUSION OF TREATMENT OF 2ND RELAPSE AND THE BEGINNING OF TREATMENT OF 3RD RELAPSE):

. . . . . . . . . . . . . . . . . . . . . . . . . . . . . (weeks)

TYPE OF PROGRESSION:

❑ local relapse

❑ distant metastasis

**14**

❑ Surgical procedure

LEEP/LLETZ ❑ electroconization ❑ conization ❑ hysterectomy ❑

TIME SPENT IN THE UNIT: days

TIME SPENT IN THE OPERATING THEATRE: min.

❑ Brachytherapy

❑ EBT:

❑ CHT:

combined radiation and chemotherapy ❑ radiation therapy ❑

type of chemotherapy . . . . . . . . . . . . . . . . . . . . . . . . . . . . . . . . . . . . . . . . . . . . . . . . . . . .

. . . . . . . . . . . . . . . . . . . . . . . . . . . . . . . . . . . . . . . . . . . . . . . . . . . . . . . . . . . . . . . . . .

. . . . . . . . . . . . . . . .

number of cycles: . . . . . . . . . .

reasons for discontinuation of treatment:
remission ❑ adverse reactions ❑ stabilization ❑ progression ❑

Date of beginning of treatment of 3rd relapse: . . . . . . . . . . . . . . . . . . . . .


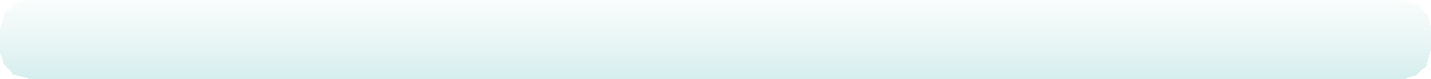


**TREATMENT OF 3RD RELAPSE**

Date of conclusion of treatment of 3rd relapse: . . . . . . . . . . . . . . . . . . . . . **15**

COMPLICATIONS


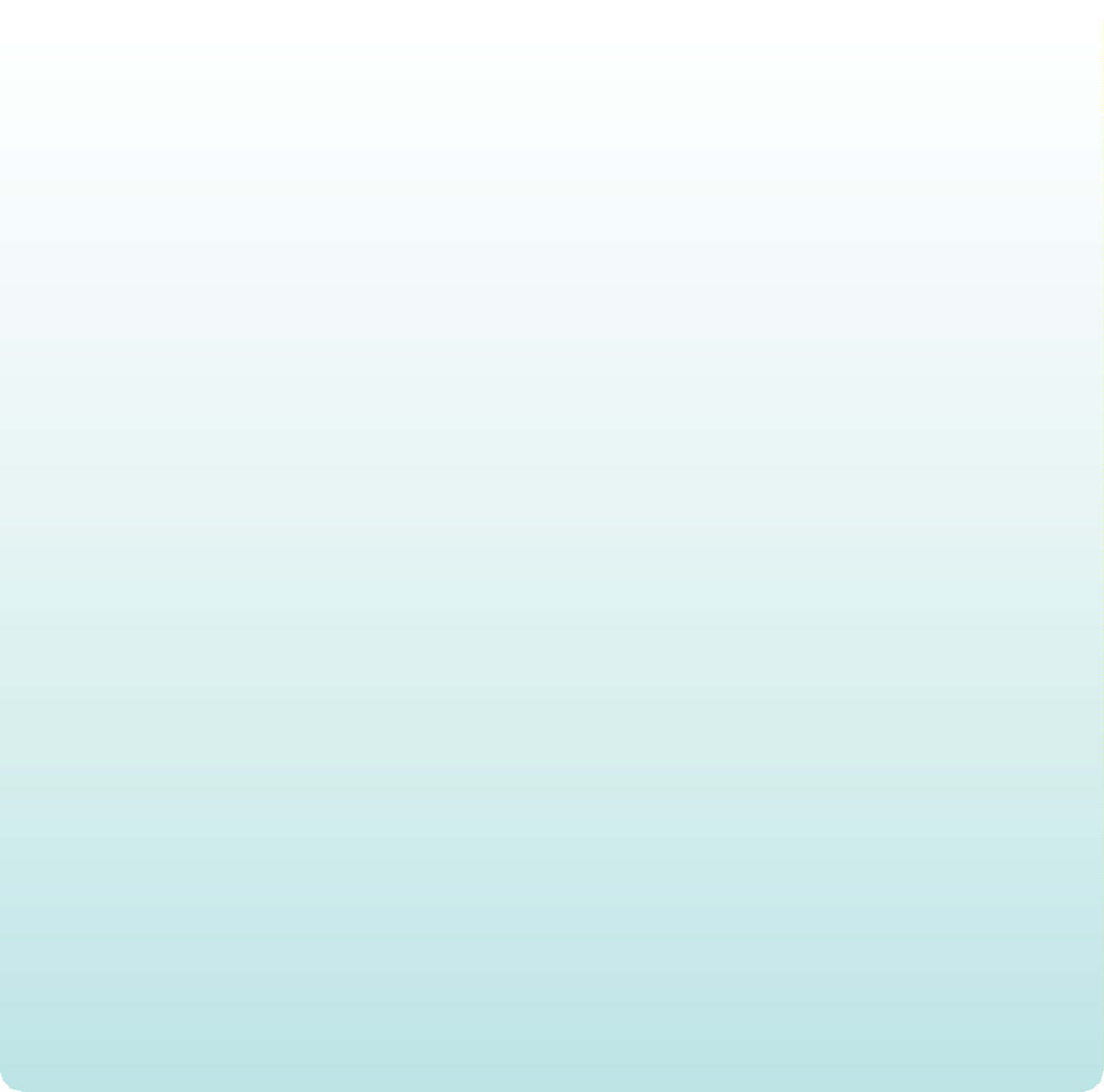

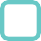


❑ fistula

type of fistula: . . . . . . . . . . . . . . . . . . . . . . . . . . . . . . . . . . . . . . . . . . . . . . . . . . . . . . . . . . . . . .

date of fistula surgery: . . . . . . . . . . . . . . .

❑ haematological complications

treatment applied: . . . . . . . . . . . . . . . . . . . . . . . . . . . . . . . . . . . . . . . . . . . . . . . . . . . . . . . . . .

. . . . . . . . . . . . . . . . . . . . . . . . . . . . . . . . . . . . . . . . . . . . . . . . . . . . . . . . . . . . . . . . . . . . . . . . .

❑ nephrological complications

treatment applied: . . . . . . . . . . . . . . . . . . . . . . . . . . . . . . . . . . . . . . . . . . . . . . . . . . . . . . . . . .

. . . . . . . . . . . . . . . . . . . . . . . . . . . . . . . . . . . . . . . . . . . . . . . . . . . . . . . . . . . . . . . . . . . . . . . . .

❑ hepatic complications

treatment applied: . . . . . . . . . . . . . . . . . . . . . . . . . . . . . . . . . . . . . . . . . . . . . . . . . . . . . . . . . .

. . . . . . . . . . . . . . . . . . . . . . . . . . . . . . . . . . . . . . . . . . . . . . . . . . . . . . . . . . . . . . . . . . . . . . . . .

❑ cardiovascular complications

treatment applied: . . . . . . . . . . . . . . . . . . . . . . . . . . . . . . . . . . . . . . . . . . . . . . . . . . . . . . . . . .

. . . . . . . . . . . . . . . . . . . . . . . . . . . . . . . . . . . . . . . . . . . . . . . . . . . . . . . . . . . . . . . . . . . . . . . . .

❑ neurological complications

treatment applied: . . . . . . . . . . . . . . . . . . . . . . . . . . . . . . . . . . . . . . . . . . . . . . . . . . . . . . . . . .

. . . . . . . . . . . . . . . . . . . . . . . . . . . . . . . . . . . . . . . . . . . . . . . . . . . . . . . . . . . . . . . . . . . . . . . . .

❑ gastrointestinal complications

treatment applied: . . . . . . . . . . . . . . . . . . . . . . . . . . . . . . . . . . . . . . . . . . . . . . . . . . . . . . . . . .

. . . . . . . . . . . . . . . . . . . . . . . . . . . . . . . . . . . . . . . . . . . . . . . . . . . . . . . . . . . . . . . . . . . . . . . . .

❑ lymphedema

treatment applied: . . . . . . . . . . . . . . . . . . . . . . . . . . . . . . . . . . . . . . . . . . . . . . . . . . . . . . . . . .

. . . . . . . . . . . . . . . . . . . . . . . . . . . . . . . . . . . . . . . . . . . . . . . . . . . . . . . . . . . . . . . . . . . . . . . . .

**16**


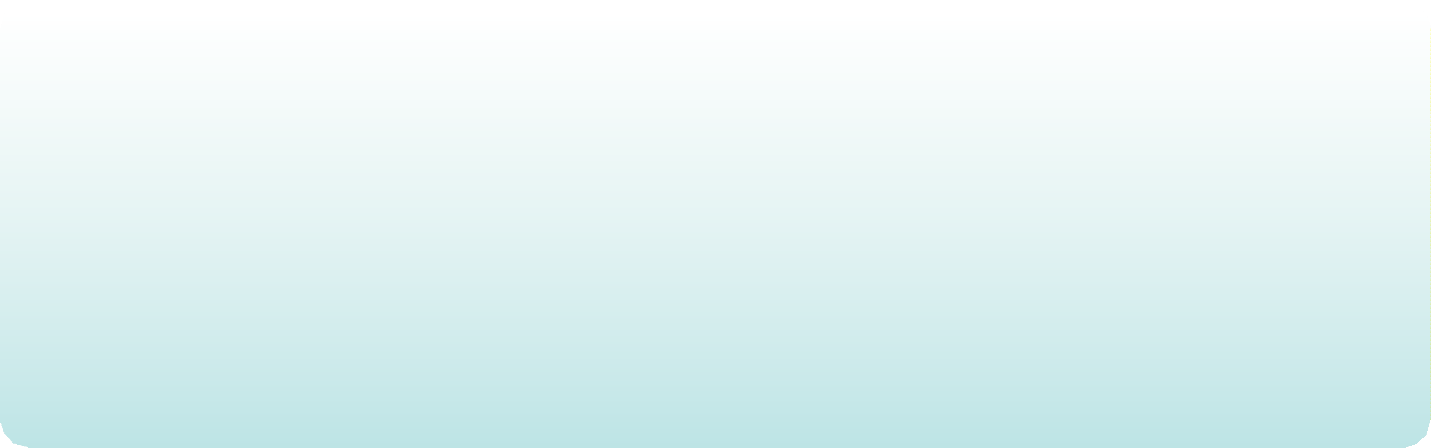

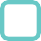


IMAGING TESTS DONE DURING DIAGNOSIS AND TREATMENT OF 3RD RELAPSE

❑ CT

❑ Ultrasonography

❑ MRI

❑ Scintigraphy

❑ PET

❑ Colposcopy

number: . . . . . . . .

number: . . . . . . . .

number: . . . . . . . .

number: . . . . . . . .

number: . . . . . . . .

number: . . . . . . . .

**17**
