# Supplementary material for: Current management of cervical cancer in Poland—Analysis of the questionnaire trial for the years 2002-2014 in relation to ASCO 2016 recommendations
Source: PLoS One. 2019 Jan 31;14(1):e0209901. doi: 10.1371/journal.pone.0209901 (PMC6354992; doi:10.1371/journal.pone.0209901)
Supplement: S2 File — (PDF) [file pone.0209901.s002.pdf]

POLSKIE TOWARZYSTWO GINEKOLOGII ONKOLOGICZNEJ

BADANIE DZIENNICZKOWE RAKA SZYJKI MACICY

SEKCJA PTGO DS. PROFILAKTYKI I LECZENIA RAKA SZYJKI MACICY

NUMER OŚRODKA I PACJENTKI ☐☐ ☐☐☐

nr ośrodka nr pacjentki

1

ZAAWANSOWANIE CHOROBY

IA ❑ IB ❑ IIA ❑ IIB ❑ IIIA ❑ IIIB ❑ IVA ❑ IVB ❑

TYP HISTOLOGICZNY NOWOTWORU: . . . .

WSPÓŁISTNIENIE CIĄŻY (w trakcie leczenia oraz do 12 miesięcy po zakończeniu) TAK ❑ NIE ❑

KLASYFIKACJA JPG: . . . .

2

RODZAJ LECZENIA PIERWOTNEGO

❑ Zabieg chirurgiczny

LEEP/LETZ ❑ elektrokonizacja ❑ konizacja ❑ usunięcie narządu rodnego ❑

CZAS POBYTU NA ODDZIALE: . . . . dni

CZAS POBYTU NA SALI OPERACYJNEJ . . . . min.

❑ Brachyterapia

❑ EBT:

radiochemioterapia ❑ radioterapia ❑

❑ CHT:

rodzaj chemioterapii . . . .

liczba cykli: . . . .

powody zakończenia leczenia: remisja ❑ działania niepożądane ❑ stabilizacja ❑ progresja ❑

Data rozpoczęcia leczenia pierwotnego: . . . .

Data zakończenia leczenia pierwotnego: . . . .

3

POWIKŁANIA

❏ przetoka

rodzaj przetoki: . . . .

data operacji przetoki: . . . .

❏ powikłania hematologiczne

zastosowane leczenie: . . . .

. . . .

❏ powikłania nefrologiczne

zastosowane leczenie: . . . .

. . . .

❏ powikłania hepatologiczne

zastosowane leczenie: . . . .

. . . .

❏ powikłania ze strony układu krążenia

zastosowane leczenie: . . . .

. . . .

❏ powikłania neurologiczne

zastosowane leczenie: . . . .

. . . .

❏ powikłania pokarmowe

zastosowane leczenie: . . . .

. . . .

❏ obrzęki limfatyczne

zastosowane leczenie: . . . .

. . . .

4

BADANIA OBRAZOWE W TRAKCIE DIAGNOSTYKI I LECZENIA PIERWOTNEGO

❏ TK liczba: . . . .

❏ USG liczba: . . . .

❏ rezonans liczba: . . . .

❏ scyntygrafia liczba: . . . .

❏ PET liczba: . . . .

❏ kolposkopia liczba: . . . .

5

LECZENIA WZNOWY I

TREATMENT FREE SURVIVAL (CZAS OD ZAKOŃCZENIA LECZENIA PIERWOTNEGO DO ROZPOCZĘCIA LECZENIA WZNOWY) :

. . . . (tygodnie)

RODZAJ PROGRESJI:

❏ wznowa miejscowa

❏ przerzuty odległe

6

RODZAJ LECZENIA WZNOWY I

❏ Zabieg chirurgiczny

LEEP/LETZ ❏ elektrokonizacja ❏ konizacja ❏ usunięcie narządu rodnego ❏

CZAS POBYTU NA ODDZIALE: . . . . dni

CZAS POBYTU NA SALI OPERACYJNEJ . . . . min.

❏ Brachyterapia

❏ EBT:

radiochemioterapia ❏ radioterapia ❏

❏ CHT:

rodzaj chemioterapii . . . .

. . . .

. . . .

liczba cykli: . . . .

powody zakończenia leczenia: remisja ❏ działania niepożądane ❏ stabilizacja ❏ progresja ❏

Data rozpoczęcia leczenia wznowy I: . . . .

Data zakończenia leczenia wznowy I: . . . .

7

POWIKŁANIA

❑ przetoka

rodzaj przetoki: . . . .

data operacji przetoki: . . . .

❑ powikłania hematologiczne

zastosowane leczenie: . . . .

. . . .

❑ powikłania nefrologiczne

zastosowane leczenie: . . . .

. . . .

❑ powikłania hepatologiczne

zastosowane leczenie: . . . .

. . . .

❑ powikłania ze strony układu krążenia

zastosowane leczenie: . . . .

. . . .

❑ powikłania neurologiczne

zastosowane leczenie: . . . .

. . . .

❑ powikłania pokarmowe

zastosowane leczenie: . . . .

. . . .

❑ obrzęki limfatyczne

zastosowane leczenie: . . . .

. . . .

BADANIA OBRAZOWE W TRAKCIE DIAGNOSTYKI I LECZENIA WZNOWY

❑ TK	liczba: . . . .
❑ USG	liczba: . . . .
❑ rezonans	liczba: . . . .
❑ scyntygrafia	liczba: . . . .
❑ PET	liczba: . . . .
❑ kolposkopia	liczba: . . . .

LECZENIA WZNOWY II

TREATMENT FREE SURVIVAL (CZAS OD ZAKOŃCZENIA LECZENIA WZNOAWY I DO ROZPOCZĘCIA LECZENIA WZNOWY II) :

. . . . (tygodnie)

RODZAJ PROGRESJI:

❏ wznowa miejscowa

❏ przerzuty odległe

10

RODZAJ LECZENIA WZNOWY II

❏ Zabieg chirurgiczny

LEEP/LETZ ❏ elektrokonizacja ❏ konizacja ❏ usunięcie narządu rodnego ❏

CZAS POBYTU NA ODDZIALE: . . . . dni

CZAS POBYTU NA SALI OPERACYJNEJ . . . . min.

❏ Brachyterapia

❏ EBT:

radiochemioterapia ❏ radioterapia ❏

❏ CHT:

rodzaj chemioterapii . . . .

. . . .

. . . .

liczba cykli: . . . .

powody zakończenia leczenia: remisja ❏ działania niepożądane ❏ stabilizacja ❏ progresja ❏

Data rozpoczęcia leczenia wznowy II: . . . .

Data zakończenia leczenia wznowy II: . . . .

11

POWIKŁANIA

❏ przetoka
rodzaj przetoki: . . . .
data operacji przetoki: . . . .

❏ powikłania hematologiczne
zastosowane leczenie: . . . .
. . . .

❏ powikłania nefrologiczne
zastosowane leczenie: . . . .
. . . .

❏ powikłania hepatologiczne
zastosowane leczenie: . . . .
. . . .

❏ powikłania ze strony układu krążenia
zastosowane leczenie: . . . .
. . . .

❏ powikłania neurologiczne
zastosowane leczenie: . . . .
. . . .

❏ powikłania pokarmowe
zastosowane leczenie: . . . .
. . . .

❏ obrzęki limfatyczne
zastosowane leczenie: . . . .
. . . .

BADANIA OBRAZOWE W TRAKCIE DIAGNOSTYKI I LECZENIA WZNOWY II

❏ TK liczba: . . . .
❏ USG liczba: . . . .
❏ rezonans liczba: . . . .
❏ scyntygrafia liczba: . . . .
❏ PET liczba: . . . .
❏ kolposkopia liczba: . . . .

LECZENIA WZNOWY III

TREATMENT FREE SURVIVAL (CZAS OD ZAKOŃCZENIA LECZENIA WZNOAWYII DO ROZPOCZĘCIA LECZENIA WZNOWY III) :

. . . . (tygodnie)

RODZAJ PROGRESJI:

❏ wznowa miejscowa

❏ przerzuty odległe

14

RODZAJ LECZENIA WZNOWY III

❏ Zabieg chirurgiczny

LEEP/LETZ ❏ elektrokonizacja ❏ konizacja ❏ usunięcie narządu rodnego ❏

CZAS POBYTU NA ODDZIALE: . . . . dni

CZAS POBYTU NA SALI OPERACYJNEJ . . . . min.

❏ Brachyterapia

❏ EBT:

radiochemioterapia ❏ radioterapia ❏

❏ CHT:

rodzaj chemioterapii . . . .

. . . .

. . . .

liczba cykli: . . . .

powody zakończenia leczenia: remisja ❏ działania niepożądane ❏ stabilizacja ❏ progresja ❏

Data rozpoczęcia leczenia wznowy III: . . . .

Data zakończenia leczenia wznowy III: . . . .

15

POWIKŁANIA

❑ przetoka

rodzaj przetoki: . . . .

data operacji przetoki: . . . .

❑ powikłania hematologiczne

zastosowane leczenie: . . . .

. . . .

❑ powikłania nefrologiczne

zastosowane leczenie: . . . .

. . . .

❑ powikłania hepatologiczne

zastosowane leczenie: . . . .

. . . .

❑ powikłania ze strony układu krążenia

zastosowane leczenie: . . . .

. . . .

❑ powikłania neurologiczne

zastosowane leczenie: . . . .

. . . .

❑ powikłania pokarmowe

zastosowane leczenie: . . . .

. . . .

❑ obrzęki limfatyczne

zastosowane leczenie: . . . .

. . . .

BADANIA OBRAZOWE W TRAKCIE DIAGNOSTYKI I LECZENIA WZNOWY III

❑ TK liczba: . . . .

❑ USG liczba: . . . .

❑ rezonans liczba: . . . .

❑ scyntygrafia liczba: . . . .

❑ PET liczba: . . . .

❑ kolposkopia liczba: . . . .
