# Supplementary material for: Current management of cervical cancer in Poland—Analysis of the questionnaire trial for the years 2002-2014 in relation to ASCO 2016 recommendations
Source: PLoS One. 2019 Jan 31;14(1):e0209901. doi: 10.1371/journal.pone.0209901 (PMC6354992; doi:10.1371/journal.pone.0209901)
Supplement: S3 File — (DOCX) [file pone.0209901.s003.docx]

**Current management of cervical cancer in Poland – analysis of the questionnaire trial for the years 2002-2014 in relation to ASCO 2016 recommendations.**

Tomasz Basta1, Paweł Blecharz2, Lubomir Bodnar3, Iwona Gawron1, Dorota Babczyk1, Magdalena Piróg1, Tomasz Kluz4, Anna Markowska5, Paweł Knapp^6^, Anna Horbaczewska1, Robert Jach1,

*1 Department of Gynecology and Obstetrics, Jagiellonian University Medical College, 23 Kopernika str., 31-501 Krakow, Poland
2 Gynecologic Oncology Department, Centre of Oncology, Maria Sklodowska-Curie Memorial Institute, Krakow Branch, Krakow, Poland.*

*3 Department of Clinical Oncology, Military Institute of Medicine in Warsaw, 128 Szaserów Str., 04-141 Warsaw, Poland
4 Department of Obstetrics and Gynecology, Fryderyk Chopin University Hospital No 1, Faculty of Medicine, Rzeszow University, Rzeszow, Poland, Poland.*

*5 Department of Perinatology and Gynecology, Poznan University of Medical Sciences, Poznan, Poland*

*6Department of Gynecology and Gynecological Oncology, Faculty of Medicine, Medical University in Bialystok, Poland*


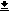


This work was supported by National Center of Science (NCN grant no. N407 152740).

**Objectives**: To assess the survival of patients with cervical cancer (CC). Since the recommendations concerning cervical cancer management adopted by Polish medical societies do not differ significantly from the ESGO or non-European guidelines and the fact that evaluation of the system for CC treatment in Poland, as well as the mortality rate of polish women with CC, which is 70% higher than the average for European Union (EU) countries, justifies the hypothesis that treatment of CC in Poland deviates from the Polish and international recommendations. This article put forward the current management of cervical cancer in Poland and discusses it in the context of ASCO guidelines.

**Material and methods**: A survey retrospective multicenter analysis of the medical records of 1247 patients with cervical cancer who underwent treatment for disease and who had completed at least **two** years of follow-up.

**Results:** Although concurrent radiotherapy and chemotherapy is a standard treatment of FIGO IB to IVA cervical cancer patients in enhanced- and maximal-resources settings in our analysis we found that the percentage of women subjected to chemotherapy was lower than in countries where total survival rates are lower.

**Conclusion**: Within the IA to II A cervical cancer patients studied group the methods of treatment remained with agreement with ASCO guidelines for countries with the highest standard of care.
Although concurrent radiotherapy and chemotherapy is a standard treatment of FIGO IB to IVA cervical cancer patients in enhanced- and maximal-resources settings in our analysis we found that the percentage of women subjected to chemotherapy was lower than in countries where total survival rates are lower.

Our findings together with the inconsistences within the cervical cancer screening program may be one of the explanation of poorer survival rate of women with cervical cancer in Poland.

Key Words: Cervical cancer, surgery, radiotherapy chemoradiotherapy, survey, ASCO 2016

**Introduction**

Cervical cancer (CC) is the fourth most common cancer among women. In 2012 the worldwide number of women with CC was around 528 000, of whom 266 000 died. CC in 85% of cases are diagnosed in developing countries where CC is a leading cause of death among women suffering from cancer [1].

A significant difference in incidence and mortality due to CC is observed between low- and middle-income countries and high-income countries [2, 3, 4].
According to World Bank data, Poland is one of the high-income countries [5]. However, results of CC treatment are unsatisfactory. GLOBOCAN 2012 project places Poland among countries of the highest rate of incidence and mortality due to CC with the standardized incidence rate (SIR) at 12.2/100 000 and the standardized death rate (SDR) at 5.4/100,000 (one of the highest among the surveyed countries). Both SIR and CDR are relatively high compared to other European countries [6].

According to the World Health Organization (WHO), therapeutic options in cervical cancer patients should be selected in agreement with international, national or institutional guidelines based on the combination of evidence, availability of trained professionals, equipment facilities and infrastructure [7].
To provide evidence-based and resource-stratified global recommendations on the management and palliative care of women with invasive CC, the American Society of Clinical Oncology (ASCO) convened a multidisciplinary multinational panel of experts who produced recommendations reflecting settings diversity. The aim was to develop guidelines that would provide the best medical care to patients with CC, and that would be adapted to four levels of financial resources at the same time [8].
Up to the 1990s the standard treatment of CC at the stage of IIB-IVA according to International Federation of Gynecology and Obstetrics (FIGO), or in earlier stages with histological factors of unfavorable prognosis, was the use of radiotherapy alone. A rapid increase in the incidence of combination therapy (radio- and chemotherapy) was observed since the mid 90’s of the twentieth century [9].
Extensive multicenter randomized clinical trials (RCTs) [10,11,12] demonstrated extended survival in patients with advanced CC following radiotherapy with concurrent chemotherapy based on cisplatin compared to radiotherapy alone. On the other hand, recommended management of less advanced stages of CC (FIGO I-IIA), including surgery with or without adjuvant therapy, has not undergone major changes in the last few decades.

The recommendations adopted by Polish medical societies [13, 14] do not differ significantly from the FIGO [15] or non-European guidelines [16,17].

Evaluation of the system for CC treatment in Poland [18], as well as the mortality rate of polish women with CC, which is 70% higher than the average for European Union (EU) countries [19], justifies the hypothesis that treatment of CC in Poland deviates from the Polish and international recommendations.

**Objectives**

This article put forward the current management of cervical cancer in Poland and discusses it in the context of ASCO guidelines.

**Material and Methods**

The retrospective survey study was conducted in the years 2015-2016. The inclusion criteria were women treated due to CC, who had at least **two** year of post treatment follow-up data. Cases were eligible irrespective of surgical approach, surgery type or primary treatment

Questionnaires were sent to 24 centers of gynecological oncology providing comprehensive treatment of CC. Completed questionnaires were sent back from16 centers (Szpital Uniwersytecki w Krakowie, Centrum Onkologii im M. Skłodowskiej-Curie, Świętokrzyskie Centrum Onkologii, Wojewódzki Szpital Zespolony w Kielcach, Szpital Specjalistyczny w Brzozowie, Uniwersytecki Szpital Kliniczny w Białymstoku, Białostocki Ośrodek Onkologiczny, Wielkopolskie Centrum Onkologii, Szpital Kliniczny UM w Poznaniu, Szpital Kliniczny w Lublinie, Gdyńskie Centrum Onkologii, Dolnośląskie Centrum Onkologii, Mazowiecki Szpital Bródnowski, Wojskowy Instytut Medyczny, Szpital Specjalistyczny im. M. Kopernika w Łodzi, Centrum Onkologii w Gliwicach). Of these, 4 centers entered data from 􏰀 200 patients, 10 centers 25-40 patients, and 2 centers 􏰁 25 patients. Data were collected from 1371 women treated due to a CC between 2002 and 2014.

The database was designed to capture data including: clinical staging, primary treatment, complications, imaging, treatment free survival to the first recurrence, first relapse treatment, complications and imaging associated with this management, treatment free survival to the second recurrence, second relapse treatment, complications and imaging associated with this management, treatment free survival to the third recurrence, third relapse treatment,

complications and imaging associated with this management, was designed to track the treatment of individuals with CC.
All data were reviewed by a main and senior author. Any discrepancies in data were corrected through communication between the study monitor and the individual center. Due to missing essential data, 124 patients were excluded from the database. Finally, data from 1247 patients were analyzed.

*Statistical analysis*

Data were expressed as percentage, mean 􏰂 standard deviation or median (interquartile range) unless otherwise stated. The Kolmogorov-Smirnov test was used to assess conformity with a normal distribution.
A 2-sided p-value of less than 0.05 was considered statistically significant. Statistical analyses were performed with the Statistica 12 (Statsoft, Tulsa, OK, USA).

**Results**

Patient age ranged from 27–78, with a median age of 54.
The median follow-up after diagnosis was 61 (60–67) months. Among 1218 women squamous cell cancer was diagnosed in 1070 women (87.9 %), adenocarcinoma in 112 (9.2 %), adenosquamosum in 4 (0.3%) and other types in 32 (2.6 %). Grading of cervical cancer based on histopathological examination was: G1 -14 (7.8%), G2 – 115 (64.3%) and G3 – 50 (27.9%). The distribution clinical stage was described in Table 1.

Table 1. Clinical stage of cervical cancer according to the FIGO classification.

| FIGO stage | N (1145) | (%) |
| --- | --- | --- |
| IA | 74 | 6.5 |
| IB | 353 | 30.8 |
| IIA | 123 | 10.7 |
| IIB | 289 | 25.2 |
| IIIA | 19 | 1.7 |
| IIIB | 229 | 20.0 |
| IVA | 20 | 1.8 |
| IVB | 38 | 3.3 |

First-line treatment

In the first-line therapy surgery was performed in 540 (**43**%) women (507 cases of radical hysterectomy, (**95** %); ~~10 cases of cervical conization,~~ **~~2~~**~~%; 11 cases of cervical conization with subsequent radical hysterectomy,~~ **~~2~~**~~%; 5 cases of loop electrosurgical excision procedure/ Large loop excision of the transformation zone, LEEP/LEETZ,~~ **~~1~~**~~% of cases).~~ **15 cases of conization, 3% of cases; 11 cases of cervical conization with subsequent radical hysterectomy, 2%.** Brachytherapy (BT) was performed in 889 (**71**%) women and teleradiotherapy (with or without chemotherapy) in 871 (**70**%) women included in the study (radiotherapy in 194 women, **16**%; radiochemotherapy in 677 women, **54**%; Detailed analysis of treatment methods combinations is presented in Table 2.

**Table 2**. Combinations of treatment methods in the first-line therapy of cervical cancer

| First-line treatment | N (%) | Additional BT  n (% after first-line treatment) | Additional RT  n (% after first-line treatment) | Additional CT  n (% after first-line treatment) |
| --- | --- | --- | --- | --- |
| Cc | 16 (1,2%) | 1 (6,3%) | 2 (12,5%) | 3 (18,8%) |
| Cc+RH | 10 (0,8%) | 1 (10%) | - | - |
| RH | 507 (40,7%) | 347 (64,3%) | 240 (47,3%) | 188 (37,1%) |
| BT | 540 (43,3%) | - | 518 (95,9%) | 463 (85,7%) |
| RT | 111 (8,9%) | - | - | 43 (38,7%) |
| CT | 30 (2,4%) | - | - | - |
| No treatment | 33 (7,7%) | - | - | - |

Abbreviations: LEEP; loop electrosurgical excision procedure, LLETZ; large loop excision of the transformation zone; RH, radical hysterectomy; Cc, cervical conization; BT, brachytherapy; RT, radiotherapy; CT, chemotherapy; RCT, radiochemotherapy;


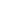


Chemotherapy was applied in 727 (58.30%) cases. The most commonly used drug was cisplatin alone (50%), less frequently cisplatin in combination with 5-fluorouracil (21%) and carboplatin with paclitaxel (16%) (Table ~~5~~ **3)**.

Table 3. Chemotherapy used in first-line treatment of cervical cancer.

| Chemotherapy | N (191) | % |
| --- | --- | --- |
| cisplatin | 98 | 51.3 |
| cisplatin and 5-fluorouracil | 42 | 22.0 |
| paclitaxel and carboplatin | 31 | 16.2 |
| cispltin and topotecan | 5 | 2.6 |
| cisplatin and paclitaxel | 2 | 1.0 |
| cisplatin and carboplatin | 2 | 1.0 |
| cisplatin and gemcitabine | 1 | 0.5 |
| topotecan and indykcyna? | 1 | 0.5 |
| combinations of schemes | 9 | 4.7 |
|  |  |  |

Progression after primary treatment was found in 9.3% of cases. Local recurrence and distal metastases occurred in patients treated with brachytherapy in combination with radiotherapy and brachytherapy in combination with radiochemotherapy, and concerned stages FIGO IB and IIB. **Type of cervical cancer recurrence was dependent on the first-line treatment. (Table 5.)**

Table 5. Type of cervical cancer recurrence depending on the first-line treatment.

| Treatment method | Local recurrence  N (42) | Distant metastases  N (86) |
| --- | --- | --- |
| No treatment | 1 | 2 |
| RH | 1 | 0 |
| RH + BT | 3 | 1 |
| RH + RT | 2 | 0 |
| RH+ RCT | 0 | 2 |
| RH + BT+ RT | 2 | 2 |
| RH + BT+ CT | 4 | 6 |
| RH + BT + RCT | 4 | 6 |
| LEEP/LLETZ + BT + RCT | 1 | 0 |
| LEEP/LLETZ + RCT | 1 | 0 |
| BT + CT | 0 | 1 |
| BT + RT | 1 | 6 |
| BT | 1 | 0 |
| BT + RCT | 17 | 55 |
| RCT | 3 | 5 |
| RT | 1 | 0 |

Abbreviations: see Table 2

Distal metastases were more frequent (67.24%) than local recurrence (31.03%) (Table 6).

Table 6. Number of cases of cervical cancer recurrence after first-line treatment in relation to FIGO stage

| FIGO stage | Local recurrence | Distant metastases |
| --- | --- | --- |
| IA | 2 | 0 |
| IB | 8 | 11 |
| IIA | 4 | 15 |
| IIB | 15 | 30 |
| IIIA | 2 | 3 |
| IIIB | 7 | 17 |
| IVA | 0 | 1 |
| IVB | 0 | 3 |

The highest percentage of local recurrences and distant metastases was found in the group treated with brachytherapy (14.7%) in combination with radiochemotherapy (47.41%) (Table 7) and FIGO IIB (12.93%) with distant metastases (25.86%) (Table 8).

Table 7. Cervical cancer recurrence treatment (Second line therapy)

| Recurrence treatment | N (116) | % |
| --- | --- | --- |
| No further treatment | 25 | 21.5 |
| RH | 2 | 1.7 |
| RH + CT | 2 | 1.7 |
| RH + RCT | 1 | 0.9 |
| Cc + CT | 1 | 0.9 |
| Cc + BT + RCT | 1 | 0.9 |
| BT | 3 | 2.6 |
| BT + RCT | 1 | 0.9 |
| RT | 15 | 12.8 |
| CT | 55 | 47.4 |
| RCT | 9 | 7.8 |
| Cc | 1 | 0.9 |

Abbreviations: see Table 2

Table 8. Chemotherapy used in recurrence treatment

| Treatment | N (76) | (%) |
| --- | --- | --- |
| paclitaxel and carboplatin | 21 | 27.6 |
| cisplatin and 5-fluorouracil | 17 | 22.4 |
| cisplatin and topotecan | 10 | 13.2 |
| cisplatin | 8 | 10.5 |
| cisplatin and paclitaxel | 6 | 7.9 |
| paclitaxel | 1 | 1.3 |
| paclitaxel and topotecan | 1 | 1.3 |
| topotecan | 1 | 1.3 |
| Ifosfamide | 1 | 1.3 |
| combination of schemes | 10 | 13.2 |

Second-line treatment

The first recurrence after primary treatment was most often treated with chemotherapy in 55 women (47%). The mean time from the end of primary treatment to the start of recurrence treatment in women included in the study was 62 weeks (95% CI: 50-74 weeks; 15.5 months, 95% CI: 12.5-18.5 months). The most common second-line management was chemotherapy (47.41%), no treatment (21.55%) and radiotherapy (12.93%) (Table 9).

Table 11. Combinations of second-line treatment.

| Second-line treatment | n | (%) |
| --- | --- | --- |
| No treatment | 25 | 21,5 |
| RH | 2 | 1,7 |
| RH+CT | 2 | 1,7 |
| RH+RCT | 1 | 0,9 |
| Cc+CT | 1 | 0,9 |
| Cc + Bt + RCT | 1 | 0,9 |
| Bt | 3 | 2,6 |
| Bt + RCT | 1 | 0,9 |
| RT | 15 | 12,9 |
| CT | 55 | 47,4 |
| RCT | 9 | 7,8 |
| Cc | 1 | 0,9 |

Abbreviations: see Table 2

The most frequent regimen used in the second-line chemotherapy was paclitaxel with carboplatin (27%), followed by cisplatin and 5-fluorouracil (22.08%) and cisplatin and topotecan (12.99%) (Table 10).

Table 10. Type of chemotherapy in second-line treatment.

| Type of chemotherapy | **n** | **(%)** |
| --- | --- | --- |
| cisplatin | 8 | 10,4 |
| cisplatin + 5-fluorouracyl | 17 | 22,0 |
| cisplatin + paclitaxel | 6 | 7,8 |
| cisplatin + topotecan | 10 | 13 |
| paclitaxel | 1 | 1,3 |
| paclitaxel + carboplatin | 21 | 27,2 |
| paclitaxel + topotecan | 1 | 1,3 |
| topotecan | 1 | 1,3 |
| ifosfamid | 1 | 1,3 |
| combination | 10 | 13 |

Discussion

The survival rate in CC patients in Poland, according to the National Health Fund (Narodowy Fundusz Zdrowia, NFZ) in the years 2005-2010 was 55%, however, according to National Cancer Registry (Krajowy Rejestr Nowotworów, KRN) in the years 2003-2005 it was **54%** and according to Eurocare in the years 1999-2007 it was **53%.** The values of comparable indicators in Poland were about 10% lower than average levels in Europe (RSC, according to Eurocare) [20]. According to NFZ data, there were significant differences in survival rates between voivodeships, reaching 20 percentage points (Podlaskie **Voivodeship** 67%, Lodzkie **Voivodeship** 45%). There were also discrepancies in these indicators, depending on the data source (NFZ data vs. KRN data), which cannot be explained but other data methodology [21].

Treatment of CC at an early stage involves surgery and radiotherapy. Surgical methods are generally reserved for stage IA, IB1, and in some cases, IIA1
Chemoradiotherapy is a method of choice in the treatment of stages IB2 to IVA. It can also be used in patients who are not eligible for surgery [22, 23].

In guidelines of leading societies (with the exeption of European society for medical oncology- ESMO) radical hysterectomy with pelvic lymphadenectomy, and para-aortic lymph node sampling in selecteded cases are recommended in women with early-stage or locally advanced disease.

**According to ESMO, radical hysterectomy is not recommended for an early stage disease. Microinvasive cervical cancer (stage IA1) without lymphovascular space invasion (LVSI) can be managed with conisation or simple trachelectomy to preserve fertility and simple hysterectomy can be offered if the patient does not wish to preserve fertility. In stage IA1 with LVSI, surgical assessment of pelvic lymph nodes should be discussed with the patient, including the sentinel lymph node.  This approach provides**

**much less mutilating procedure for the patient [24, 25, 26].**

In our study we found that in first-line therapy surgery was performed in 540 (**43**%) patients (507 cases of radical hysterectomy, 95%; ~~10 cases of cervical conization,~~ **~~2~~**~~%; 11 cases of cervical conization with subsequent radical hysterectomy, 2%; 5 cases of loop electrosurgical excision procedure / large loop excision of the transformation zone, LEEP/LEETZ,~~ **~~1~~**~~% of~~ ~~cases).~~ **15 cases of conization, 3% of cases; 11 cases of cervical conization with subsequent radical hysterectomy, 2%.** Taking into account that the group of patients at stage IA to IIA accounted for 48% of patients, this method of treatment remained with agreement with ASCO guidelines for countries with the highest standard of care. For women with locally advanced CC, the role of surgery has been debated for many years and little benefit from such management was found for most women in stages IB2 to III [27].

Concurrent radiotherapy and chemotherapy is a standard treatment in FIGO IB to IVA in enhanced- and maximal-resources settings [28, 29, 30, 31, 32, 33, 34]
In our analysis we found that brachytherapy was performed in **71 %** women and teleradiotherapy (with or without chemotherapy) in **70**% women included in the study (radiotherapy in **16**%; radiochemotherapy in **54**%).

Chemotherapy was applied in 58% cases. The most commonly used drug was cisplatin (**50**% of cases), less frequently cisplatin in combination with 5-fluorouracil (21%) and carboplatin with paclitaxel (**16**%).

In our study population the percentage of women subjected to chemotherapy was lower than in countries where total survival rates are lower **[35].** Perhaps this observation resulted from efforts not to delay the use of radiotherapy as a result of administrating chemotherapy.
In 16% of women chemotherapy scheme consisted of carboplatin and paclitaxel, which proves the management of high-income settings, as depicted in ongoing clinical trial [36]. This trial is investigating the influence of the addition of adjuvant carboplatin and paclitaxel after chemoradiotherapy versus concurrent cisplatin and radiotherapy in patients of FIGO IB to IVA. An additional phase III trial of concurrent chemotherapy and pelvic irradiation with or without adjuvant carboplatin and paclitaxel is open for high-risk patients with stage IA2, IB, or IIA cervical carcinoma after radical hysterectomy [37].

Strengths of the study

This was the first study investigating CC management in the Polish population. The observation time in this study was longer than reported in other studies [38], nevertheless the mean follow- up was 5.5 years.

Limitations of the study

As other population studies based on administrative data [39,40], the study has some limitations related to the availability of the data. Therefore, we could only assess tumor-related factors, management, recurrence-free survival. We could not take into account additional individual factors, in particular the effects of comorbidities, general health condition and preferences in the treatment. Similarly, we could not evaluate other factors related to medical practice such as local practice of referring patients to an oncology center, potential limitations in healthcare access and patient’s adherence. Therefore, we are not sure how each of these uncontrolled factors contributed to the observed outcomes.

Tracing the extent to which the clinical practice coincides with the recommendations is to be a field of future research.

Conclusions
Within the IA to II A cervical cancer patients studied group the methods of treatment remained with agreement with ASCO guidelines for countries with the highest standard of care. Although concurrent radiotherapy and chemotherapy is a standard treatment of FIGO IB to IVA cervical cancer patients in enhanced- and maximal-resources settings in our analysis we found that the percentage of women subjected to chemotherapy was lower than in countries where total survival rates are lower.
Our findings together with the inconsistences within the cervical cancer screening program may be one of the explanation of poorer survival rate of women with cervical cancer in Poland.

References

[1] International Agency for Research on Cancer: GLOBOCAN 2012 Cervical Cancer: Estimated Incidence, Mortality and Prevalence Worldwide in 2012. http://globocan.iarc.fr/old/FactSheets/cancers/cervix-new.asp.
[2] Olson B, Gribble B, Dias J, Curryer C, Vo K, Kowal P, Byles J. Cervical cancer screening programs and guidelines in low- and middle-income countries. Int J Gynaecol Obstet. 2016; 134: 239-46
[3] Gelband H., Jha P., Sankaranarayanan R., Horton S. Cancer. Disease Control Priorities, third edition, vol. 3, International Bank for Reconstruction and Development / The World Bank, Washington, DC 20433, USA, 2015
[4] Randall T.C., Ghebre R. Challenges in prevention and care delivery for women with cervical cancer in Sub-Saharan Africa. Front. Oncol. 2016; 28:160

[5]  http://data.worldbank.org/country/poland

[6]  Ferlay J, Soerjomataram I, Dikshit R, Eser S, Mathers C, Rebelo M, Parkin DM, Forman

D, Bray F. Cancer incidence and mortality worldwide: sources, methods and major patterns in GLOBOCAN 2012. Int J Cancer. 2015; 136: 359-386
[7] World Health Organization. Comprehensive Cervical Cancer Control: a guide to essential practice-2nd edition. World Health Organization, Geneva 2014, Switzerland
[8] Chuang L.T., Temin S., Berek J.S. Management and care of women with invasive cervical cancer: american society of clinical oncology resource-stratified clinical practice guideline summary. J. Oncol. Pract. 2016; 12: 693–696
[9] Thomas GM. Improved treatment for cervical cancer concurrent chemotherapy and radiotherapy. N Eng J Med. 1999; 340: 1198-1200
[10] Keys HM, Bundy BN, Stehman FB, Muderspach LI, Chafe WE, Suggs CL, 3rd, et al. Cisplatin, radiation, and adjuvant hysterectomy compared with radiation and adjuvant hysterectomy for bulky stage IB cervical carcinoma. New Engl J Med. 1999; 340: 1154–61 [11] Morris M, Eifel PJ, Lu J, Grigsby PW, Levenback C, Stevens RE, et al. Pelvic radiation with concurrent chemotherapy compared with pelvic and para-aortic radiation for high-risk cervical cancer. New Engl J Med. 1999; 340: 1137–43
[12] Rose PG, Bundy BN, Watkins EB, Thigpen JT, Deppe G, Maiman MA, et al. Concurrent cisplatin-based radiotherapy and chemotherapy for locally advanced cervical cancer. New Engl J Med. 1999; 340: 1144–53


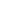

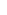

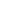

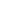

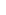


[13] Nasierowska-Guttmejer A, Kędzia W, Wojtylak S, Lange D, Rokita W, Jach R, Wielgoś M. Polish recommendations regarding diagnostics and treatment of cervical squamous intraepithelial lesions according to the CAP/ASCCP guidelines. Ginekol Pol. 2016; 87: 676-682

[14] Nowak-Markwitz E, Basta A, Kotarski J, Markowska J, Oszukowski P, Sajdak S, Sawicki W, Spaczyński M; Polish Gynecological Society. Systemic therapy of recurrent or persistent cancer of the cervix. Recommendations of the Polish Gynecological Society. Ginekol Pol. 2015; 86: 712-4

[15] PecorelliS, ZiglianiL, OdicinoF. Revised FIGO staging for carcinoma of the cervix. Int J Gynaecol Obstet 2009; 105: 107–8
[16] Small W Jr, Strauss JB, Jhingran A, Yashar CM, Cardenes HR, Erickson-Wittmann BA, Gullett N, Kidd E, Lee LJ, Mayr NA, Moore D, Puthawala AA, Rao GG, Varia MA, Wahl AO, Wolfson AH, Yuh W, Gaffney DK. ACR Appropriateness Criteria® definitive therapy for early-stage cervical cancer. Am J Clin Oncol. 2012; 35: 399-405

[17] Siegel CL, Andreotti RF, Cardenes HR, Brown DL, Gaffney DK, Horowitz NS, Javitt MC, Lee SI, Mitchell DG, Moore DH, et al. ACR Appropriateness Criteria® pretreatment planning of invasive cancer of the cervix. J Am Coll Radiol. 2012; 9: 395-402
[18] Jach R, Blecharz P, Kozierkiewicz A. Rak szyjki macicy-diagnoza systemu. Polskie Towarzystwo Ginekologii Onkologicznej. Sekcja ds. profilaktyki i leczenia raka szyjki macicy PTGO. Warszawa, 2016

[19] http://onkologia.org.pl/nowotwory-szyjki-macicy-kobiet/
[20] De Angelis R, Sant M, Coleman MP, Francisci S, Baili P, Pierannunzio D, Trama A, Visser O, Brenner H, Ardanaz E, Bielska-Lasota M, Engholm G, Nennecke A, Siesling S, Berrino F, Capocaccia R; EUROCARE-5 Working Group. Cancer survival in Europe 1999- 2007 by country and age: results of EUROCARE--5-a population-based study. Lancet Oncol. 2014; 15: 23-34
[21]. Nowakowski A, Wojciechowska U, Wieszczy P, Cybulski M, Kamiński MF, Didkowska J. Trends in cervical cancer incidence and mortality in Poland: is there an impact of the introduction of the organised screening? Eur J Epidemiol. 2017 Jun;32(6):529-532
[22] ACOG practice bulletin. Diagnosis and treatment of cervical carcinomas, number 35, May 2002. Obstet Gynecol. 2002;99: 855-67
[23] Wiebe E, Denny L, Thomas G. Cancer of the cervix uteri. Int J Gynaecol Obstet. 2012; 119 Suppl 2: S100-9


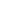

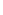

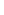


[24] Koh WJ, Greer BE, Abu-Rustum NR, Apte SM, Campos SM, Cho KR, Chu C, Cohn D, Crispens MA, Dorigo O, Eifel PJ, Fisher CM, Frederick P, Gaffney DK, Han E, Huh WK, Lurain JR 3rd, Mutch D, Fader AN, Remmenga SW, Reynolds RK, Teng N, Tillmanns T, Valea FA, Yashar CM, McMillian NR, Scavone JL. Cervical Cancer, Version 2.2015. J Natl Compr Canc Netw. 2015; 13: 395-404

[25] Marth C, Landoni S, Mahner S, McCormack M, Gonzalez-Martin A, Colombo N Cervical cancer: ESMO Clinical Practice Guidelines for diagnosis, treatment and follow-up. Ann Oncol 2017;28:72–83.
[26] Ebina Y, Yaegashi N, Katabuchi H, Nagase S, Udagawa Y, Hachisuga T, Saito T, Mikami M, Aoki Y, Yoshikawa H. Japan Society of Gynecologic Oncology guidelines 2011 for the treatment of uterine cervical cancer. Int J Clin Oncol. 2015; 20: 240-8

[27] Kokka F, Bryant A, Brockbank E, Powell M, Oram D. Hysterectomy with radiotherapy or chemotherapy or both for women with locally advanced cervical cancer. Cochrane Database Syst Rev. 2015; 7: CD010260
[28] Morris M, Eifel PJ, Lu J, et al.: Pelvic radiation with concurrent chemotherapy compared with pelvic and para-aortic radiation for high-risk cervical cancer. N Engl J Med 1999; 340: 1137-43

[29] Keys HM, Bundy BN, Stehman FB, et al.: Cisplatin, radiation, and adjuvant hysterectomy compared with radiation and adjuvant hysterectomy for bulky stage IB cervical carcinoma. N Engl J Med 1999; 340: 1154-61
[30] Peters WA 3rd, Liu PY, Barrett RJ 2nd, et al.: Concurrent chemotherapy and pelvic radiation therapy compared with pelvic radiation therapy alone as adjuvant therapy after radical surgery in high-risk early-stage cancer of the cervix. J Clin Oncol 2000; 18: 1606-13

[31] Thomas GM: Improved treatment for cervical cancer--concurrent chemotherapy and radiotherapy. N Engl J Med 2000; 340: 1198-200
[32] Pearcey R, Brundage M, Drouin P, et al.: Phase III trial comparing radical radiotherapy with and without cisplatin chemotherapy in patients with advanced squamous cell cancer of the cervix. J Clin Oncol 2002; 20: 966-7

[33] Rose PG, Bundy BN: Chemoradiation for locally advanced cervical cancer: does it help? J Clin Oncol 2002; 20: 891-3, 2002
[34] Chemoradiotherapy for Cervical Cancer Meta-Analysis Collaboration: Reducing uncertainties about the effects of chemoradiotherapy for cervical cancer: a systematic review and meta-analysis of individual patient data from 18 randomized trials. J Clin Oncol 2008; 26: 5802-12

[35] Bodurka-Bevers D, Morris M, Eifel PJ, et al. Posttherapy surveillance of women with cervical cancer: an outcomes analysis. Gynecol Oncol 2000;78:187-193.

[36] Zuliani AC, Esteves SC, Teixeira LC, Teixeira JC, de Souza GA, Sarian LO. Concomitant cisplatin plus radiotherapy and high-dose rate brachytherapy versus radiotherapy alone for stage IIIB epidermoid cervical cancer: a randomized
controlled trial. J Clin Oncol. 2014; 32: 542-7

[37] Srivastava K, Paul S, Chufal KS, Shamsundar SD, Lal P, Pant MC, Bhatt M, Singh S, Gupta R. Concurrent chemoradiation versus radiotherapy alone in cervical carcinoma: A randomized phase III trial. Asia Pac J Clin Oncol. 2013; 9: 349-56

[38] Kowalska JD, Wroblewska A, Ząbek P, Firląg-Burkacka E, Kalinowska M, Byczot Z, Horban A Barriers to cervical cancer screening exist despite integrating HIV and gynaecological services for HIV-positive women in Poland. Ginekol Pol. 2018;89(2):68-73 [39] Age-specific differences in the treatment of cervical cancer in the east and the south of The Netherlands 1989-2004.van der Aa MA, Siesling S, v d Poll-Franse LV, Schutter EM, Lybeert ML, Coebergh JW Eur J Obstet Gynecol Reprod Biol. 2009; 147: 78-82

[40] Impact of adoption of chemoradiotherapy on the outcome of cervical cancer in Ontario: results of a population-based cohort study. Pearcey R, Miao Q, Kong W, Zhang-Salomons J, Mackillop WJ. J Clin Oncol. 2007; 25: 2383-8
