# Supplementary material for: Current management of cervical cancer in Poland—Analysis of the questionnaire trial for the years 2002-2014 in relation to ASCO 2016 recommendations
Source: PLoS One. 2019 Jan 31;14(1):e0209901. doi: 10.1371/journal.pone.0209901 (PMC6354992; doi:10.1371/journal.pone.0209901)
Supplement: S4 File — (DOC) [file pone.0209901.s004.doc]

Opis statystyczny – cała grupa –1247 przypadków

Pacjentki przebywające w szpitalu w latach: 2002 - 2014

Zdecydowaną większość przypadków stanowiły raki płaskonabłonkowe szyjki macicy (88%), następnie raki gruczołowe (9%) i rak gruczołowy z metaplazją nabłonkową (0,3%). Pozostały odsetek zajmują inne nowotwory: lymphoepithelioma, clarocellulare, microcellulare, macrocellulare, sarkoma, papillary squamotransitional, solidum, keratodes, endometrioides.

Table A. Rodzaj nowotworu szyjki macicy na podstawie badania histopatologicznego.

| **Rodzaj nowotworu** | **n** | **odsetek (%)** |
| --- | --- | --- |
| Ca. planoepitheliale | 1070 | 87,85 |
| Adenocarcinoma | 112 | 9,2 |
| Adenosquamosum | 4 | 0,33 |
| inne | 32 | 2,63 |

Najwięcej przypadków nowotworów zarejestrowano w stopniu zróżnicowania G2 (64%), najmniej w stopniu G1 (8%).

Table B. Stopień zróżnicowania nowotworów szyjki macicy na podstawie badania histopatologicznego.

| **Grading** | **n** | **odsetek (%)** |
| --- | --- | --- |
| G1 | 14 | 7,82 |
| G2 | 115 | 64,25 |
| G3 | 50 | 27,93 |

Większości nowotworów nadano stopień IB zaawansowania klinicznego (31%), następnie IIB (25%) i IIIB (20%). Najmniejszy odsetek nowotworów dotyczył stopnia zaawansowania klinicznego IIIA (1,66%) i IVA (1,75%) przypadków.

Table C. Stopień zaawansowania klinicznego nowotworów szyjki macicy.

| **zaawansowanie nowotworu** | **n** | **odsetek (%)** |
| --- | --- | --- |
| IA | 74 | 6,46 |
| IB | 353 | 30,83 |
| IIA | 123 | 10,74 |
| IIB | 289 | 25,24 |
| IIIA | 19 | 1,66 |
| IIIB | 229 | 20,00 |
| IVA | 20 | 1,75 |
| IVB | 38 | 3,32 |

I LINIA LECZENIA

Zabieg chirurgiczny wykonano u 540 (43,3%) pacjentek (usunięcie narządu rodnego 507 - 95,12% przypadków, konizacja 10 – 1,87%, konizacja z usunięciem narządy rodnego 11 – 2,03%, LEEP/LEETZ – 5 – 0,94% przypadków). Leczenie brachyterapią przeprowadzono u 889 (71,29%) a teleradioterapię (z lub bez chemioterapii) u 871 (69,85%) kobiet włączonych do badania (radioterapia – 194 – 15,56%, radiochemioterapia – 677 – 54,29%). Chemioterapię zastosowano w 727 (58,30%) przypadkach – najczęściej - 50% cisplatyna.

Progresję stwierdzono w 9,3% przypadków. Wznowa miejscowa i przerzuty odległe wystąpiły w dwóch przypadkach: leczenia brachyterapią i radioterapią oraz brachyterapią i radiochemioterapią; oraz w stopniu zaawansowania IB i IIB.

Table D. Rodzajj progresji po leczeniu pierwotnym.

| **Rodzaj progresji** | **n** | **odsetek (%)** |
| --- | --- | --- |
| przerzuty odległe | 78 | 67,24 |
| wznowa miejscowa | 36 | 31,03 |
| przerzuty odległe + wznowa miejscowa | 2 | 1,72 |

Table E. Rodzaj wznowy w zależności od leczenia I linii.

| **Kombinacje leczenia** | **Wznowa miejscowa** | **Przerzuty odległe** |
| --- | --- | --- |
| Bez leczenia | 1 | 2 |
| Usunięcie narządu rodnego | 1 | 0 |
| Usunięcie narządu rodnego + brachyterapia | 3 | 1 |
| Usunięcie narządu rodnego + radioterapia | 2 | 0 |
| Usuniecie narządu rodnego + radiochemioterapia | 0 | 2 |
| Usunięcie narządu rodnego + brachyterapia + radioterapia | 2 | 2 |
| Usunięcie narządu rodnego + brachyterapia + chemioterapia | 0 | 0 |
| Usunięcie narządu rodnego + brachyterapia + radiochemioterapia | 4 | 6 |
| LEEP/LEETZ + brachyterapia + radiochemioterapia | 1 | 0 |
| LEEP?LEETZ + radiochemioterapia | 1 | 0 |
| Brachyterapia + chemioterapia | 0 | 1 |
| Brachyterapia + radioterapia | 1 | 6 |
| Brachyterapia | 1 | 0 |
| Brachyterapia + radiochemioterapia | 17 | 55 |
| Radiochemioterapia | 3 | 5 |
| Radioterapia | 1 | 0 |

Table F. Liczba przypadków wznowy po I linii leczenia w zależności od stopnia zaawansowania klinicznego.

|  | **wznowa miejscowa** | **przerzuty odległe** |
| --- | --- | --- |
| IA | 2 | 0 |
| IB | 8 | 11 |
| IIA | 4 | 15 |
| IIB | 15 | 30 |
| IIIA | 2 | 3 |
| IIIB | 7 | 17 |
| IVA | 0 | 1 |
| IVB | 0 | 3 |

Największy odsetek wznowy miejscowej i przerzutów odległych stwierdzono w przypadku zastosowania brachyterapii z radiochemioterapią (odpowiednio 17 przypadków – 14,66% i 55 przypadków – 47,41%) oraz w stopniu zaawansowania IIB (15 przypadków – 12,93% -wznowy miejscowej i 30 przypadków – 25,86% - przerzutów odległych).

Przetokę stwierdzono w 3,85% przypadków po pierwszej linii leczenia.

Wśród nich największy odsetek stanowiły przetoki odbytniczo-pochwowe (ok. 52% stwierdzonych przetok) i pęcherzowo-pochwowe (31% przypadków).

Table G. Rodzaj przetoki stwierdzonej po pierwszej linii leczenia.

| **Rodzaj przetoki** | **n** | **odsetek (%)** |
| --- | --- | --- |
| jelitowo-pochwowa | 2 | 4,17 |
| odbytniczo-pochwowa | 25 | 52,08 |
| odbyniczo pochwowa i pęcherzowo-pochwowa | 3 | 6,25 |
| odbytniczo-pochwowa i moczowodowo-pochwowa | 1 | 2,08 |
| moczowodowo-pochwowa | 1 | 2,08 |
| pęcherzowo-pochwowa | 15 | 31,25 |

Największą liczbę przetok stwierdzono w stopniu zaawansowania IIB (40% przypadków przetoki) oraz IIIB (23% przypadków).

Table H. Rodzaj przetoki w zależności od zaawansowania klinicznego nowotworu.

| **Rodzaj przetoki** | **IA** | **IB** | **IIA** | **IIB** | **IIIA** | **IIIB** | **IVA** | **IVB** |
| --- | --- | --- | --- | --- | --- | --- | --- | --- |
| jelitowo-pochwowa | 0 | 0 | 0 | 1 | 0 | 0 | 0 | 0 |
| odbytniczo-pochwowa | 1 | 2 | 3 | 11 | 0 | 6 | 1 | 0 |
| odbyniczo pochwowa i pęcherzowo-pochwowa | 0 | 0 | 1 | 1 | 0 | 1 | 0 | 0 |
| odbytniczo-pochwowa i moczowodowo-pochwowa | 0 | 0 | 0 | 1 | 0 | 0 | 0 | 0 |
| moczowodowo-pochwowa | 0 | 0 | 0 | 1 | 0 | 0 | 0 | 0 |
| pęcherzowo-pochwowa | 0 | 2 | 1 | 4 | 0 | 5 | 2 | 1 |

Powikłania po leczeniu pierwotnym stwierdzono w 23,65% przypadków

Table I. Powikłania po leczeniu pierwotnym.

| **Rodzaj powikłań** | **n** | **Odsetek (%)** |
| --- | --- | --- |
| hematologiczne | 142 | 11,39 |
| nefrologiczne | 46 | 3,69 |
| hepatologiczne | 10 | 0,8 |
| związane z układem krążenia | 22 | 1,76 |
| neurologiczne | 14 | 1,12 |
| pokarmowe | 54 | 4,33 |
| obrzęki limfatyczne | 7 | 0,56 |

Średni czas od zakończenia leczenia I linii do rozpoczęcia leczenia wznowy w grupie kobiet włączonych do badania wynosi 62tyg (95% PU 50-74 tyg) = 15,5mc (95% PU 12,5-18,5 mc).

II LINIA LECZENIA

Wznowę po leczeniu pierwotnym leczono najczęściej chemioterapią (55 przypadków – 47%).

Table J. Kombinacja leczenia w II linii.

| **Sposób leczenia wznowy = II linia leczenia** | **n** | **Odsetek (%)** |
| --- | --- | --- |
| bez dalszego leczenia | 25 | 21,55 |
| usunięcie narządu rodnego | 2 | 1,72 |
| usunięcie narządu rodnego + chemioterapia | 2 | 1,72 |
| Usunięcie narządu rodnego + radiochemioterapia | 1 | 0,86 |
| konizacja + chemioterapia | 1 | 0,86 |
| Konizacja + brachyterapia + radiochemioterapia | 1 | 0,86 |
| brachyerapia | 3 | 2,59 |
| Brachyterapia + radiochemioterapia | 1 | 0,86 |
| radioterapia | 15 | 12,93 |
| chemioterapia | 55 | 47,41 |
| radiochemioterapia | 9 | 7,76 |
| LEEP/LEETZ | 1 | 0,86 |

Głównym schematem chemioterapii stosowanym w II linii leczenia był paklitaksel z karboplatyną (27% przypadków).

Method of treatment. N (1247)

Po zastosowaniu leczenia II linii remisję uzyskano w 13% przypadków, stabilizację w 22% przypadków, natomiast dalszą progresję w 45% przypadków (przerzuty odległe w 58% przypadków, wznowa miejscowa 33% przypadków, wznowa miejscowa i towarzyszące przerzuty odległe w 8% przypadków).

Stwierdzono 4 przypadkki przetok po leczeniu II linii: 2 przypadki przetoki odbytniczo-pochwowej, 1 przypadek pęcherzowo pochwowej i 1 przypadek jelitowo-pochwowej.

Powikłania ze strony innych narządów wystąpiły w 49% przypadków.

Table K. Rodzaj stwierdzonych powikłań po II linii leczenia.

| **Rodzaj powikłań** | **n** | **Odsetek (%)** |
| --- | --- | --- |
| hematologiczne | 27 | 23,28 |
| nefrologiczne | 9 | 7,76 |
| hepatologiczne | 5 | 4,3 |
| związane z układem krążenia | 3 | 2,59 |
| neurologiczne | 3 | 2,59 |
| pokarmowe | 8 | 6,9 |
| obrzęki limfatyczne | 2 | 1,72 |

Średni czas od zakończenia leczenia II linii do rozpoczęcia leczenia wznowy w grupie kobiet włączonych do badania wynosi 85tyg (95% PU 2-167 tyg) przedział ufności średniej zbyt szeroki – niemiarodajny, wynika z małej próby – 12 przypadków.

Analiza III linii leczenia zawiera dane o 3 przypadkach – zbyt mało danych do analizy.

**Szpital Uniwersytecki** na tle całości grupy badanych – 211 przypadków.

Pacjentki przebywające w szpitalu w latach: 2002 - 2014

Zdecydowaną większość przypadków stanowiły raki płaskonabłonkowe szyjki macicy (84%), następnie raki gruczołowe (8%) i rak gruczołowy z metaplazją nabłonkową (1,5%). Pozostały odsetek zajmują inne nowotwory: lymphoepithelioma, microcellulare, papillary squamotransitional.

Table L. Rodzaj nowotworu szyjki macicy na podstawie badania histopatologicznego.

| **Rodzaj nowotworu** | **n** | **odsetek (%)** |
| --- | --- | --- |
| Ca. planoepitheliale | 173 | 83,98 |
| Adenocarcinoma | 17 | 8,25 |
| Adenosquamosum | 3 | 1,46 |
| inne | 13 | 6,31 |

Najwięcej przypadków nowotworów zarejestrowano w stopniu zróżnicowania G2 (62,5%), najmniej w stopniu G1 (5,4%).

Table M. Stopień zróżnicowania nowotworów szyjki macicy na podstawie badania histopatologicznego.

| **Grading** | **n** | **odsetek (%)** |
| --- | --- | --- |
| G1 | 6 | 5,36 |
| G2 | 70 | 62,50 |
| G3 | 36 | 32,14 |

Większości nowotworów nadano stopień IB zaawansowania klinicznego (34%), następnie IIB (26%) i IA (25%). Najmniejszy odsetek nowotworów dotyczył stopnia zaawansowania klinicznego IIIA (1,49%) i IVA (1,49%) przypadków. Nie odnotowano żadnego przypadku w stopniu IVB zaawansowania klinicznego.

Table N. Stopień zaawansowania klinicznego nowotworów szyjki macicy.

| **zaawansowanie nowotworu** | **n** | **odsetek (%)** |
| --- | --- | --- |
| IA | 33 | 24,63 |
| IB | 45 | 33,58 |
| IIA | 12 | 8,96 |
| IIB | 35 | 26,12 |
| IIIA | 2 | 1,49 |
| IIIB | 5 | 3,73 |
| IVA | 2 | 1,49 |
| IVB | 0 | 0,00 |

I LINIA LECZENIA

Zabieg chirurgiczny wykonano u 177 (83,89%) pacjentek (usunięcie narządu rodnego 157 – 74,41% przypadków, konizacja 8 – 3,79%, konizacja z usunięciem narządy rodnego 10 – 4,74%, LEEP/LEETZ – 2 – 0,95% przypadków). Leczenie brachyterapią przeprowadzono u 83 (39,34%) a teleradioterapię (z lub bez chemioterapii) u 46 (15,17%) kobiet włączonych do badania (radioterapia – 14 – 6,64%, radiochemioterapia – 32 – 15,17%). Chemioterapię zastosowano w 48 (22,75%) przypadkach – najczęściej - 91% cisplatyna.

Table O. Kombinacje metod I linii leczenia nowotworów szyjki macicy.

| **Kombinacje leczenia** | **n** | **Odsetek (%)** |
| --- | --- | --- |
| Bez leczenia | 5 | 2,37 |
| Usunięcie narządu rodnego | 89 | 42,18 |
| Konizacja | 7 | 3,32 |
| Konizacja + usunięcie narządu rodnego | 9 | 4,27 |
| LEEP/LEETZ | 1 | 0,47 |
| Usunięcie narządu rodnego + brachyterapia | 34 | 16,11 |
| Usunięcie narządu rodnego + radioterapia | 0 | 0,00 |
| Usunięcie narządu rodnego + chemioterapia | 4 | 1,90 |
| Usuniecie narządu rodnego + radiochemioterapia | 2 | 0,95 |
| Usunięcie narządu rodnego + brachyterapia + radioterapia | 12 | 5,69 |
| Usunięcie narządu rodnego + brachyterapia + chemioterapia | 3 | 1,42 |
| Usunięcie narządu rodnego + brachyterapia + radiochemioterapia | 13 | 6,16 |
| Konizacja + chemioterapia | 1 | 0,47 |
| Usunięci narządu rodnego + konizacja + brachyterapia | 1 | 0,47 |
| LEEP/LEETZ + brachyterapia + radiochemioterapia | 1 | 0,47 |
| LEEP?LEETZ + radiochemioterapia | 0 | 0,00 |
| Brachyterapia + chemioterapia | 1 | 0,47 |
| Brachyterapia + radioterapia | 2 | 0,95 |
| Brachyterapia | 3 | 1,42 |
| Brachyterapia + radiochemioterapia | 13 | 6,16 |
| Chemioterapia | 7 | 3,32 |
| Radiochemioterapia | 3 | 1,42 |
| Radioterapia | 0 | 0,00 |

Table P. Rodzaj chemioterapii zastosowany w I linii leczenia.

| **Rodzaj chemioterapii** | **n** | **Odsetek (%)** |
| --- | --- | --- |
| cisplatyna | 40 | 90,91 |
| cisplatyna i 5-fluorouracyl | 2 | 4,55 |
| cisplatyna i topotekan | 1 | 2,27 |
| topotekan i indykcyna | 1 | 2,27 |
| kombinacja leczenia | 4 | 10,00 |

Progresję stwierdzono w 6,64% przypadków.

Table Q. Rodzajj progresji po leczeniu pierwotnym.

| **Rodzaj progresji** | **n** | **odsetek (%)** |
| --- | --- | --- |
| przerzuty odległe | 3 | 78,57 |
| wznowa miejscowa | 11 | 21,43 |

Table R. Liczba przypadków wznowy po I linii leczenia w zależności od stopnia zaawansowania klinicznego.

|  | **wznowa miejscowa** | **przerzuty odległe** |
| --- | --- | --- |
| IA | 1 | 0 |
| IB | 5 | 1 |
| IIA | 1 | 0 |
| IIB | 3 | 0 |
| IIIA | 1 | 0 |
| IIIB | 0 | 1 |
| IVA | 0 | 1 |
| IVB | 0 | 0 |

Największy odsetek wznowy miejscowej i przerzutów odległych stwierdzono w stopniu zaawansowania IB (5 przypadków – 35,71% -wznowy miejscowej i 1 przypadek – 7,14% - przerzutów odległych).

Przetokę stwierdzono w 2 (0,95%) przypadkach po pierwszej linii leczenia.

Przetoka jelitowo-pochwowa oraz przetoka jelitowo-pochwowa – obie w stopniu zaawansowania IB.

Powikłania po leczeniu pierwotnym stwierdzono w 14,68% przypadków

Table S. Powikłania po leczeniu pierwotnym.

| **Rodzaj powikłań** | **n** | **Odsetek (%)** |
| --- | --- | --- |
| hematologiczne | 10 | 4,74 |
| nefrologiczne | 4 | 1,9 |
| hepatologiczne | 1 | 0,47 |
| związane z układem krążenia | 11 | 5,21 |
| neurologiczne | 1 | 0,47 |
| pokarmowe | 3 | 1,42 |
| obrzęki limfatyczne | 1 | 0,47 |

Średni czas od zakończenia leczenia I linii do rozpoczęcia leczenia wznowy w grupie kobiet włączonych do badania wynosi 73tyg (95% PU 33-112tyg) = 18,25mc (95% PU 8,25-28mc).

II LINIA LECZENIA

Wznowę po leczeniu pierwotnym leczono najczęściej chemioterapią (7 przypadków – 50%).

Głównym schematem chemioterapii stosowanym w II linii leczenia była cisplatyna z topotekanem (44% przypadków).

Po zastosowaniu leczenia II linii remisję uzyskano w 7 przypadkach (50%), stabilizację w 2 przypadkach (14%), natomiast dalszą progresję w 5 przypadkach (36%) – 3 przypadki wznowy miejscowej i 1 przypadek wznowy miejscowej z towarzyszącymi przerzutami odległymi.

Stwierdzono 1 przypadek przetoki odbytniczo-pochwowej po leczeniu II linii.

Powikłania ze strony innych narządów wystąpiły w 64% przypadków.

Średni czas od zakończenia leczenia II linii do rozpoczęcia leczenia wznowy w grupie kobiet włączonych do badania jest niemożliwy do określenia - wynika z małej próby – 4 przypadki.

Table T. Rodzaj stwierdzonych powikłań po II linii leczenia.

| **Rodzaj powikłań** | **n** | **Odsetek (%)** |
| --- | --- | --- |
| hematologiczne | 8 | 57,14 |
| nefrologiczne | 3 | 21,43 |
| hepatologiczne | 1 | 7,14 |
| związane z układem krążenia | 2 | 14,29 |
| neurologiczne | 1 | 7,14 |
| pokarmowe | 3 | 21,43 |
| obrzęki limfatyczne | 1 | 7,14 |

Analiza III linii leczenia zawiera dane o 4 przypadkach – wszystkie leczone chemioterapią ( 3 przypadki – paklitaksel z karboplatyną, 1 przypadek cisplatyny z 5-fluorouracylem). We wszystkich przypadkach wystąpiły powikłania hematologiczne, dodatkowo w jednym z nich powikłania pokarmowe i neurologiczne.
